# Multiphysics Modeling and Analysis for Dendrite Problems in Solid-State Lithium/Sodium Metal Batteries

**DOI:** 10.1007/s40820-026-02200-0

**Published:** 2026-06-25

**Authors:** Bang Yu, Hang Su, Zengyu Yan, Yun Su, Byoungwoo Kang, Xiaohui Rong, Liquan Chen, Yong-Sheng Hu

**Affiliations:** 1 Beijing National Laboratory for Condensed Matter Physics, Institute of Physics, Chinese Academy of Sciences, Beijing, 100190 People’s Republic of China; 2 University of Chinese Academy of Sciences, Nanjing, 211135 People’s Republic of China; 3 Yangtze River Delta Physics Research Center Co. Ltd, Liyang, 213300 People’s Republic of China; 4 Chemical Defense Institute, Beijing, 100191 People’s Republic of China; 5 Department of Materials Science and Engineering, POSTECH, Pohang, Republic of Korea

**Keywords:** Solid-state batteries, Lithium/sodium metal batteries, Dendrites, Multiphysics

## Abstract

The commercialization of liquid lithium-ion batteries has revolutionized the consumer electronics industry. However, conventional lithium-ion batteries with graphite anodes and organic electrolytes are approaching their intrinsic performance limits and struggle to meet the growing demands for higher energy density, reliability, and safety in electric vehicles and large-scale energy storage. Solid-state batteries utilizing lithium or sodium metal anodes are considered promising next-generation energy storage solutions. Despite this potential, the formation of dendrites during charge–discharge cycling remains a critical challenge. Dendrite growth can initiate a destructive feedback loop of crack propagation and further dendrite intrusion, ultimately leading to battery failure and performance degradation. Previous studies have predominantly focused on single physical domains, such as electrochemical, stress, or thermal fields. However, such single-physics approach limits the understanding of dendrite evolution under realistic, coupled multiphysics conditions. This review first compares the morphological characteristics of dendrites in liquid and solid-state metal batteries. It then critically examines the key factors and predictive models of dendrite formation, initially from single-physics and subsequently from an integrated multiphysics perspective. Finally, strategies for mitigating dendrite growth via multiphysics field regulation are summarized. By establishing a comprehensive framework that integrates morphology evolution, multiphysics modeling, and suppression strategies, this work provides a foundational theoretical understanding for addressing dendrite formation in solid-state lithium and sodium metal batteries.

## Highlights

- Experimental observations of metal dendrites are systematically summarized across liquid and solid-state battery systems.
- Dendrite evolution is elucidated from a multiphysics perspective, highlighting the coupling of electrochemical, thermal, and mechanical fields.
- Mechanism-guided dendrite-suppression strategies are critically reviewed based on physical field regulation.

## Graphical abstract

**Figure:**
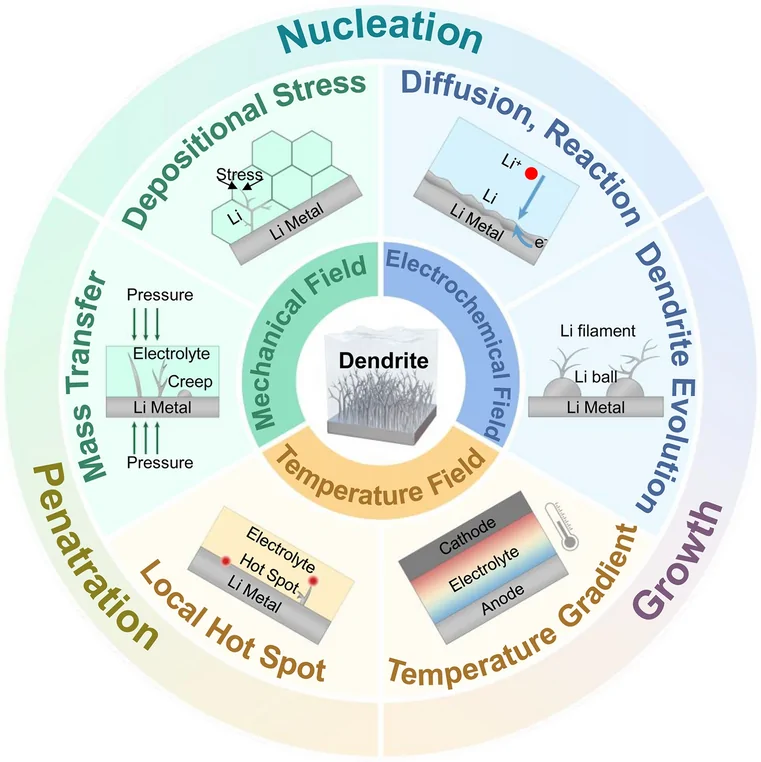

## Introduction

Since 1990s, lithium-ion batteries have emerged as the leading energy storage solution for portable electronics, enabling the widespread adoption of consumer devices [1]. However, the rise of demanding applications like electric vehicles and large-scale grid energy storage requires battery technologies that meet more rigorous standards for safety, energy density, and cycle life [2, 3]. Conventional lithium-ion battery systems employing graphite anodes and organic electrolytes are increasingly insufficient to meet these demands. Considerable research efforts are focused on developing next-generation, high-performance battery systems capable of meeting the requirements of these emerging applications. Lithium metal, as a next-generation anode, offers substantial advantages over conventional graphite [4]. It delivers an exceptionally high theoretical specific capacity (3860 vs. 372 mAh g^−1^ for graphite) and a low redox potential (− 3.04 V vs. standard hydrogen electrode (SHE)), making it a highly attractive candidate for high-energy–density batteries [5–7]. Beyond lithium, sodium metal has emerged as a promising alternative for sustainable sodium batteries, primarily owing to its natural abundance and lower cost, also showing high theoretical specific capacity (1166 mAh g^−1^) and low redox potential (− 2.71 V vs. SHE) [8, 9]. The transition to solid-state electrolytes (SSEs) is a promising solution that directly addresses the safety concerns of traditional organic electrolytes, which are prone to leakage, volatility, and flammability. The non-flammable and non-volatile properties of SSEs can significantly enhance the overall battery safety. Furthermore, the superior mechanical and electrochemical stability of solid electrolytes is essential for enabling stable, long-term cycling of reactive alkali metal anodes [10]. Studies have shown that replacing a graphite anode and liquid electrolyte with a lithium metal anode and an SSE can increase gravimetric and volumetric energy densities by up to 70% and 40%, respectively [11].

**Fig. 1:**
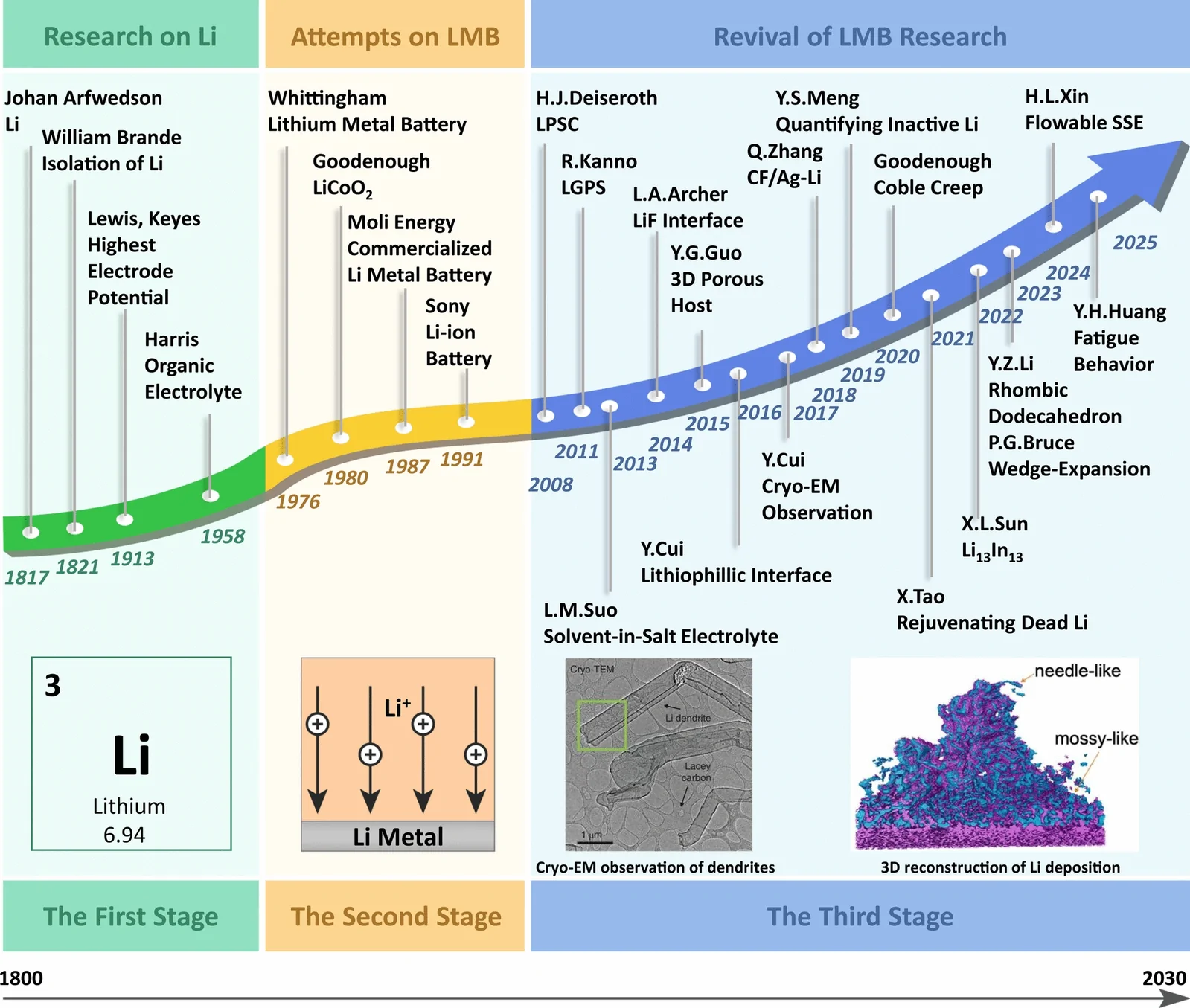
Development history of lithium metal batteries

Although battery systems employing solid electrolytes and alkali metal anodes exhibit significant application potential, several critical scientific challenges currently hinder their practical implementation [12–14]. Research on lithium metal batteries dates back to the 1950s. However, these early efforts were largely abandoned due to severe safety concerns that emerged during cycling (Fig. 1). The primary cause of these failures was the uneven deposition of metallic lithium, which led to dendritic growth. Therefore, understanding and controlling dendrite formation remain key challenges in the development of modern solid-state metal batteries [15, 16].

**Fig. 2:**
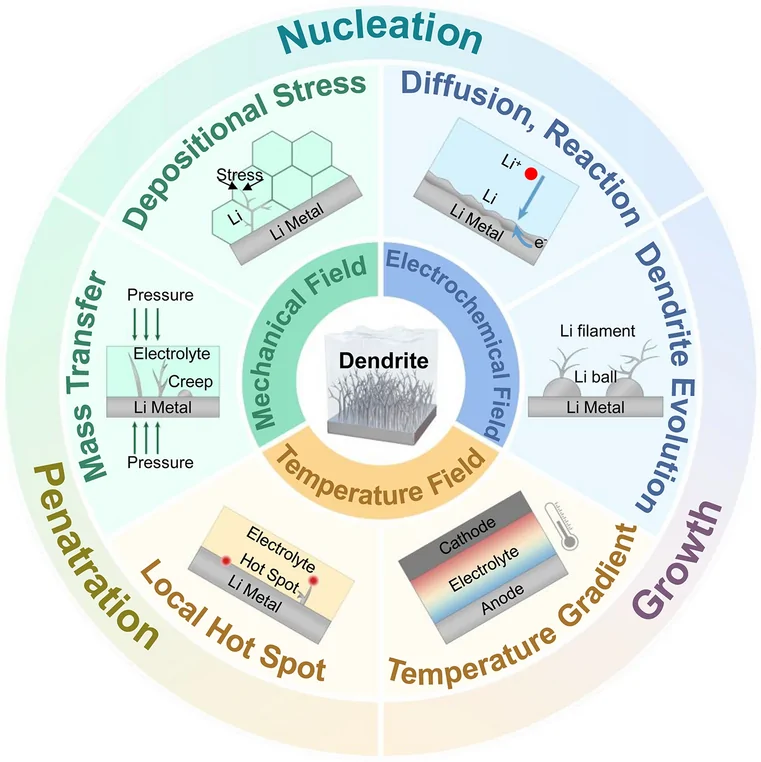
Research issues related to dendrite-associated physical fields

In liquid cells, dendrites can penetrate the separator, leading to internal short circuits. A similar failure mechanism occurs in solid-state batteries, where growing dendrites penetrate the solid electrolyte. This process initiates a destructive cycle of crack formation and accelerated dendrite growth [17], ultimately leading to performance degradation and catastrophic structure failure of the electrolyte [18, 19]. Crucially, even SSEs with a high mechanical modulus have failed to prevent dendrite-induced failure [20], underscoring the existence of a more complex propagation mechanism in solid-state systems. Much of the existing research has examined dendrite behavior from the perspectives of material modification, interface engineering, and a single physical field, such as the electrochemical, mechanical, or thermal field [3, 6, 10, 12, 14, 15, 21–23]. These studies have provided systematic and in-depth analyses of dendrite-related issues from their respective viewpoints, offering valuable insights for subsequent research. However, from the perspective of physical field mechanisms, the various physical fields inside a practical battery are not independent but are intrinsically coupled. Figure 2 briefly illustrates these interconnections. Electrochemical processes dictate local deposition kinetics, which in turn generate mechanical stresses and alter thermal distributions. Meanwhile, stress concentrations and temperature gradients feedback to modify ion transport, reaction pathways, and morphological stability. These couplings span microscopic, mesoscopic, and macroscopic scales [24], leading to dendrite growth dynamics that can deviate substantially from predictions based on single-field assumptions [25]. Therefore, a comprehensive review from a multiphysics coupling viewpoint is essential, and this is precisely the aim of the present work.

To highlight the distinct evolution characteristics of Li/Na dendrites, this review first compares their experimentally observed morphologies in liquid-state versus solid-state batteries. The focus then shifts from dendrite formation driven by individual physical fields (electrochemical, mechanical, and thermal) to the more complex mechanisms of multiphysics coupling. We critically evaluate the models describing these processes, emphasizing how the interactions and feedback mechanisms among these fields influence dendrite propagation. Building on this mechanistic understanding, we then summarize and review current strategies for suppressing dendrite growth in solid-state metal batteries. By establishing a clear framework that links morphological evolution, multiphysics models, and suppression strategies, this review aims to provide a foundational theoretical understanding for addressing the challenge of dendrite formation in solid-state Li/Na metal batteries.

## Experimental Phenomena of Li/Na Dendrite Nucleation and Growth

**Fig. 3:**
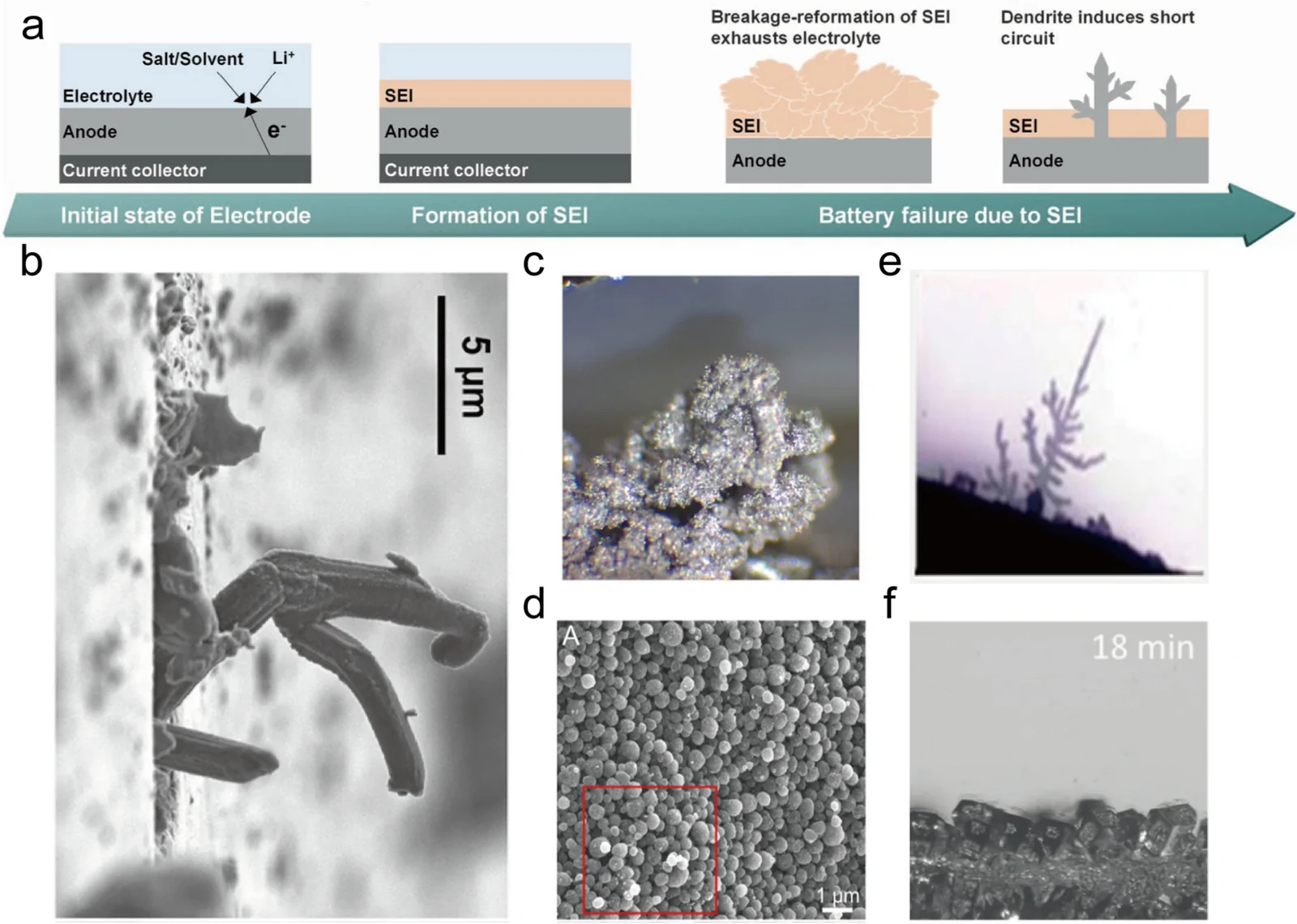
**a** Three stages of lithium deposition (Reproduced with permission from Ref. [27], Copyright 2023, Wiley). **b** Whisker-like (Reproduced with permission from Ref. [28], Copyright 2019, Elsevier); **c** moss-like (Reproduced with permission from Ref. [29], Copyright 2021, Elsevier); **d** spherical (Reproduced with permission from Ref. [30], Copyright 2021, American Chemical Society); **e** tree-like (Reproduced with permission from Ref. [23], Copyright 2021, American Chemical Society); and **f** columnar (Reproduced with permission from Ref. [31], Copyright 2018, Wiley–VCH)

The electrochemical deposition of lithium metal is typically described by three stages [26], as illustrated in Fig. 3a: (1) formation of the solid electrolyte interphase (SEI), (2) nucleation and initial growth, and (3) subsequent dendrite growth. During the initial nucleation stage, lithium ions move through the SEI and are reduced to metallic lithium at the anode surface. The density and uniformity of these initial nuclei are critical, as they determine the morphology of subsequent deposition and the propensity for dendrite formation. Following nucleation, the growth stage begins. During which, lithium atoms preferentially deposit onto the initially formed nuclei. Under specific operating or environmental conditions, these nuclei can further evolve into various dendrite structures [27]. These structures can appear in diverse forms, commonly including whisker-like, moss-like, tree-like, spherical, and columnar types (Fig. 3b–f).

### Dendrite Nucleation and Growth in Liquid Systems

#### Lithium Metal Anode System

**Fig. 4:**
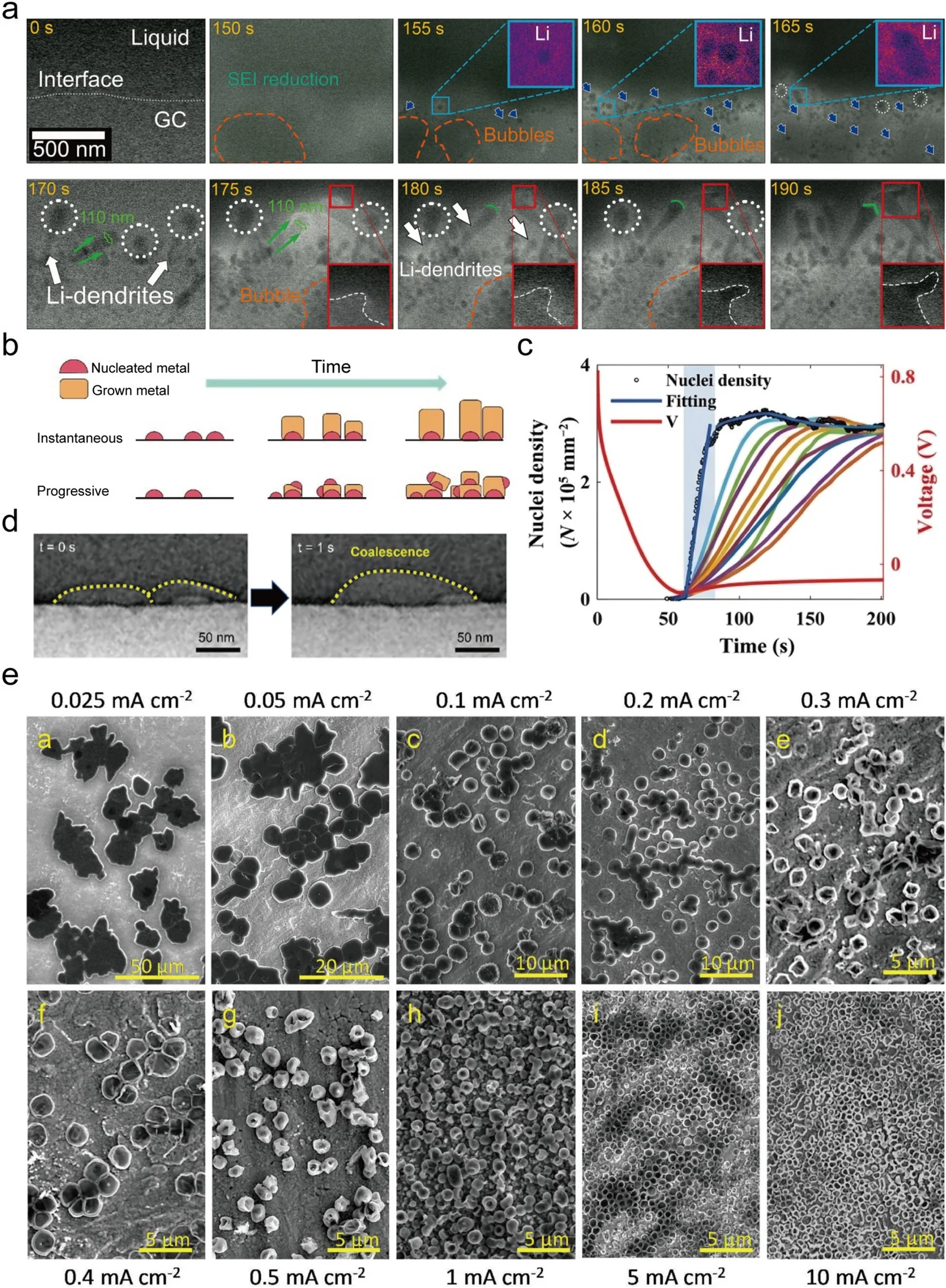
**a** Nucleation of lithium deposition at the interface (Reproduced with permission from Ref. [32], Copyright 2024, Elsevier). **b** Instantaneous and progressive nucleation modes (Reproduced with permission from Ref. [33], Copyright 2023, Wiley–VCH). **c** Nucleation density–voltage–time curve (Reproduced with permission from Ref. [34], Copyright 2023, American Association for the Advancement of Science). **d** “Liquid-like” nucleation mechanism (Reproduced with permission from Ref. [35], Copyright 2023, American Chemical Society). **e** Nucleation morphology with current density (Reproduced with permission from Ref. [38], Copyright 2017, American Chemical Society)

The initial lithium nucleation is a critical process, as the nucleation mode and its influencing factors are key determinants of the subsequent deposition morphology. As illustrated in Fig. 4a, the nucleation occurs at the anode interface beneath the SEI [32] and is generally classified into two main modes: instantaneous and progressive nucleation [33]. As illustrated in Fig. 4b, these modes are distinguished by their temporal evolution: Progressive nucleation involves the continuous formation of new nuclei over time. In contrast, instantaneous nucleation is characterized by the near-simultaneous activation of all nucleation sites, followed only by their growth. However, this distinction is not always sharply defined. Using in situ reflection interference microscopy, Feng et al. [34] observed a transitional behavior in a LiPF_6_-based electrolyte. The process initially exhibited progressive features until the nucleation rate saturated and then shifted toward an instantaneous-like nucleation mode (Fig. 4c). In addition to these classical models, Wang et al. [35] proposed a “liquid-like” nucleation mechanism via in situ TEM. This process involves the initial formation of amorphous nanoclusters that merge into liquid droplet-like nanoparticles before solidifying (Fig. 4d). Notably, this mechanism was observed prior to the formation of a distinct SEI film.

The interactions among the substrate, electrolyte, and the resulting SEI are crucial factors in controlling nucleation. Hui et al. [36] conducted a systematic investigating both substrate types (Cu substrate and Ni/LiF nanocomposite substrate) and electrolytes (LDME, perfluorocarbonate electrolyte, DOLDME, and fluorine-free carbonate electrolyte). They found that the Ni/LiF nanocomposite substrate promotes the formation of uniform, single-crystalline rhombic dodecahedral lithium in LDME and perfluorinated electrolytes, whereas the Cu substrate leads to irregular dendritic deposits. Conversely, both the Cu and Ni/LiF substrates produced dendrite-free, morphologically uniform lithium particles when DOLDME and fluorine-free carbonate electrolytes were used. This difference in nucleation morphology is closely related to the composition and characteristics of the SEI layer. Specifically, when the SEI is rich in inorganic species and has high lithium-ion conductivity, the substrate type mainly influences nucleation behavior. However, a thicker SEI or one rich in organic components hampers lithium-ion transport, rendering the nucleation process primarily controlled by the SEI. Yan et al. [37] further confirmed the fundamental role of the substrate by demonstrating a strong correlation between the nucleation overpotential and lithium solubility across different metal substrates (Au, Ag, Zn, Mg, Al, Pt, Cu, Ni, Sn, C, and Si).

Operating conditions also have a significant influence. Pei et al. [38] demonstrated that as current density increases, the nucleation density increases sharply while the nucleus size decreases, with a transition from large, island-like structures to more uniform, multilayered deposits (Fig. 4e). Quantitatively, they reported that the nucleation density increases proportionally to the cube of the current density and that critical nucleus size is inversely proportional to the current density magnitude. Furthermore, even with a fixed substrate and current density, the crystallographic orientation of the substrate can further influence nucleation density and size [39].

**Fig. 5:**
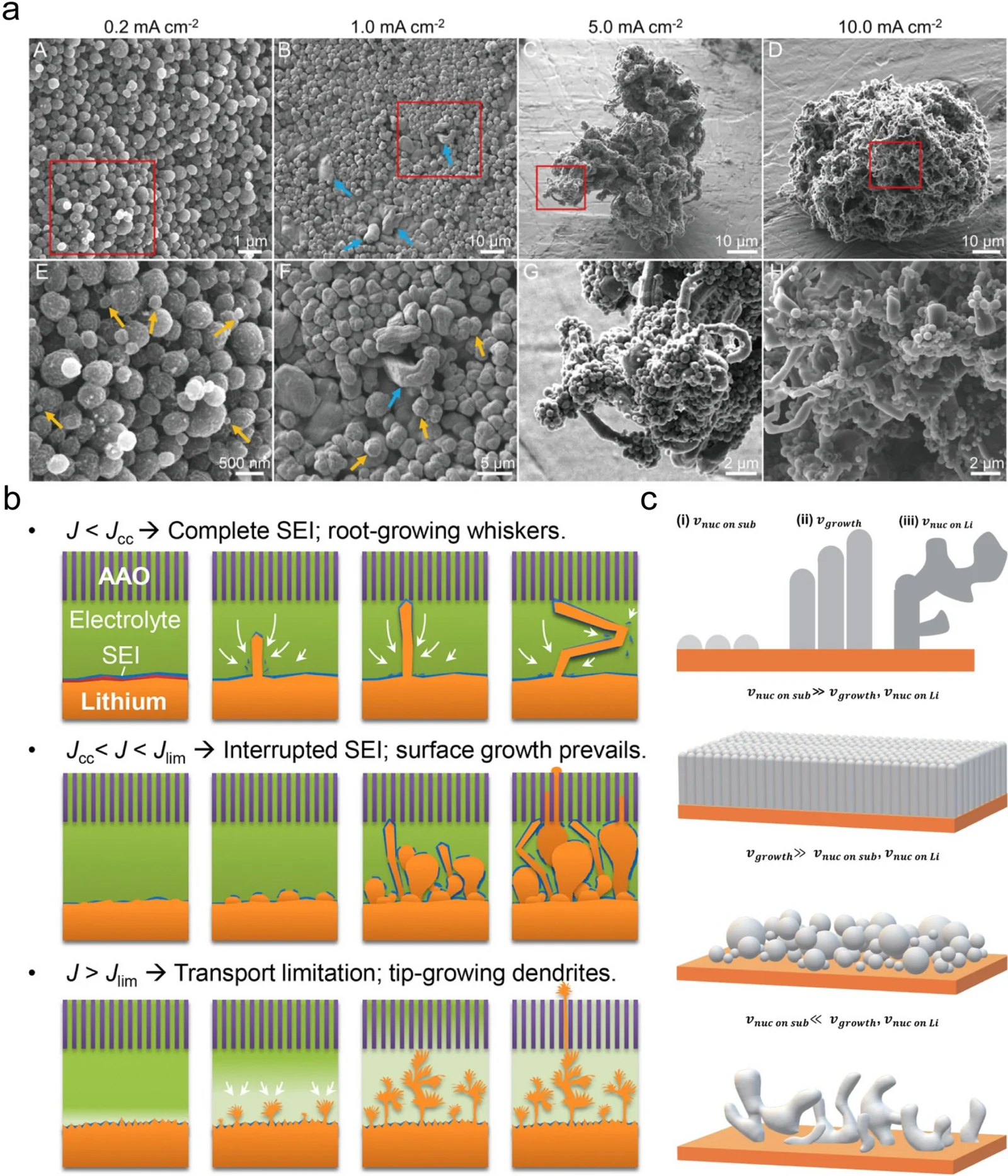
**a** Dendrite morphology with current density (Reproduced with permission from Ref. [30], Copyright 2021, American Chemical Society). **b** Relationship between current density and dendrite growth patterns (Reproduced with permission from Ref. [41], Copyright 2018, Elsevier). **c** Dendrite morphology under the influence of heterogeneous, homogeneous, and dendrite growth rates (Reproduced with permission from Ref. [45], Copyright 2022, Royal Society of Chemistry)

After nucleation, the growth and morphological evolution of lithium deposits are influenced by complex interactions among operating conditions, electrolyte chemistry, and reaction kinetics. Among these factors, current density is a primary controlling parameter. Dong et al. [30] demonstrated that in a standard LiPF_6_ (EC: DEC = 1:1 by volume) electrolyte, deposition morphology transitions from spherical at low current densities (0.2 mA cm^−2^) to columnar at medium densities (1.0 mA cm^−2^) and finally to porous, bush-like morphologies at high densities (> 5 mA cm^−2^) (Fig. 5a). Through transparent capillary cell experiments, Bai et al. [40, 41] showed that the growth and morphological evolution of lithium deposition in a LiPF_6_ (EC: DMC = 1:1 by volume) electrolyte strongly depend on current density. This dependence is manifested in the different responses of SEI formation rate and lithium deposition rate to current density [42]. As shown in Fig. 5b, dendrites grow from the root in a whisker-like pattern when the current density is below the critical value and the lithium deposition rate is lower than the SEI formation rate. The dendrites transition to a tip-growth mode exhibiting a dendritic shape when the current density exceeds the limit value and the deposition rate surpasses the SEI formation rate. Between the critical and limit current densities, the two rates are comparable, resulting in mossy surface growth. Notably, mossy and whisker-like morphologies often coexist in practice [43].

Electrolyte composition also influences morphology by modifying the interfacial ion concentration and reaction pathways. Chen et al. [44] showed that adding LiNO_3_ to a LiTFSI electrolyte can shift the morphology from whisker-like to uniform spherical particles by ensuring a sufficient Li^+^ supply beneath the SEI. However, this beneficial effect is negated at high current densities, where mass transport limitations again induced whisker growth. Furthermore, the specific combination of electrolyte salt and substrate can produce markedly different outcomes. Jo et al. [45] systematically compared the dendritic morphologies formed in LiPF_6_ (EC: DEC = 1:1) and LiFSI (EC: DEC = 1:1) electrolytes using different substrates. In the LiPF_6_ electrolyte, lithium deposited on the Cu substrate forms densely packed nuclei that grow vertically, creating a compact columnar structure. By contrast, deposition on the Ti substrate produces sparsely distributed nuclei that develop three-dimensionally into multilayered spherical lithium particles. When LiFSI is used as the electrolyte, neither dense columnar nor spherical morphologies are observed; instead, lithium rapidly nucleates on pre-deposited lithium surfaces and extends outward in a random manner, producing characteristic mossy dendrites. As shown in Fig. 5c, these morphological differences can be attributed to the interactions among the homogeneous nucleation rate (secondary nucleation on existing lithium), the heterogeneous nucleation rate (nucleation on the substrate), and the dendritic growth rate. Columnar dendrites generally form when the heterogeneous nucleation rate significantly exceeds both the homogeneous nucleation rate and the growth rate. Spherical morphologies occur when the growth rate is dominant. Conversely, mossy dendrites develop when the homogeneous nucleation rate and growth rate are significantly higher than the heterogeneous nucleation rate.

#### Sodium Metal Anode System

Research on sodium deposition and dendrite formation is often framed in comparison with the more extensively studied lithium system. Sodium exhibits similar nucleation behaviors, including progressive, instantaneous, and mixed modes [46]. The nucleation kinetics are highly sensitive to current density. Using ^23^Na NMR, Bayley et al. [47] demonstrated a transition from progressive nucleation at low current densities (0.5 mA cm^−2^) to instantaneous nucleation at higher densities (1–2 mA cm^−2^). This transition was accompanied by a dramatic increase in the specific surface area of the deposit. By defining a metric for the fraction of high-surface-area sodium (FHSA), they found that FHSA is approximately one at higher currents, indicating the formation of a loose, porous structure. The substrate also plays a critical role. Zou et al. [48] observed that sodiophilic AlSi current collectors promoted the formation of spherical particles or block-like structures. In contrast, pure Al, which lacks sodiophilic sites, resulted in a classic dendritic morphology.

Although the morphologies of sodium dendrites often resemble those of lithium dendrites [49, 50] (e.g., whisker-like or mossy), distinct differences in their growth mechanisms and structural characteristics have been identified. For instance, Song et al. [51] observed a unique cubic morphology in an inorganic NaAlCl_4_·2SO_2_ electrolyte, a morphology distinct from those observed in organic systems. More fundamentally, Hu et al. [52] identified a divergence in the growth mode: Sodium generally forms micron-sized particles through a surface-growth mechanism, whereas lithium produces submicron whiskers via a root-growth mechanism. This morphological difference stems from differences in the mechanical properties of their respective SEI layers. The mechanically robust SEI on lithium metal can withstand deposition pressure, forcing Li to extrude from the root of the structure. In contrast, the SEI on sodium is mechanically weaker and prone to continuous fracture and reformation during plating. This repeated rupture of the SEI allows sodium to deposit on the exposed surface, leading to particle accumulation instead of whisker growth.

### Dendrite Nucleation and Growth in Solid-State Systems

Although SSEs were initially expected to prevent dendrite formation owing to their high mechanical modulus, experimental evidence shows that dendrites can still grow, often at critical current densities lower than those observed in liquid systems [52]. This paradox indicates that the shear modulus alone is insufficient to predict dendrite suppression; instead, factors such as the quality of the electrode–electrolyte interface and the presence of intrinsic material defects (e.g., grain boundaries and voids) are critical [53]. Dendrite growth in solid-state batteries is a complex process, with growth mechanisms and morphologies varying considerably across different electrolyte types, such as polymers, sulfides, and oxides. Previous reviews on this topic have typically organized their analyses by electrolyte class [54, 55].

Accordingly, this section is structured not by electrolyte type, but rather by key aspects of metal deposition, including nucleation sites and mechanisms, dendrite growth modes, and morphological evolution.

#### Lithium Metal Anode System

**Fig. 6:**
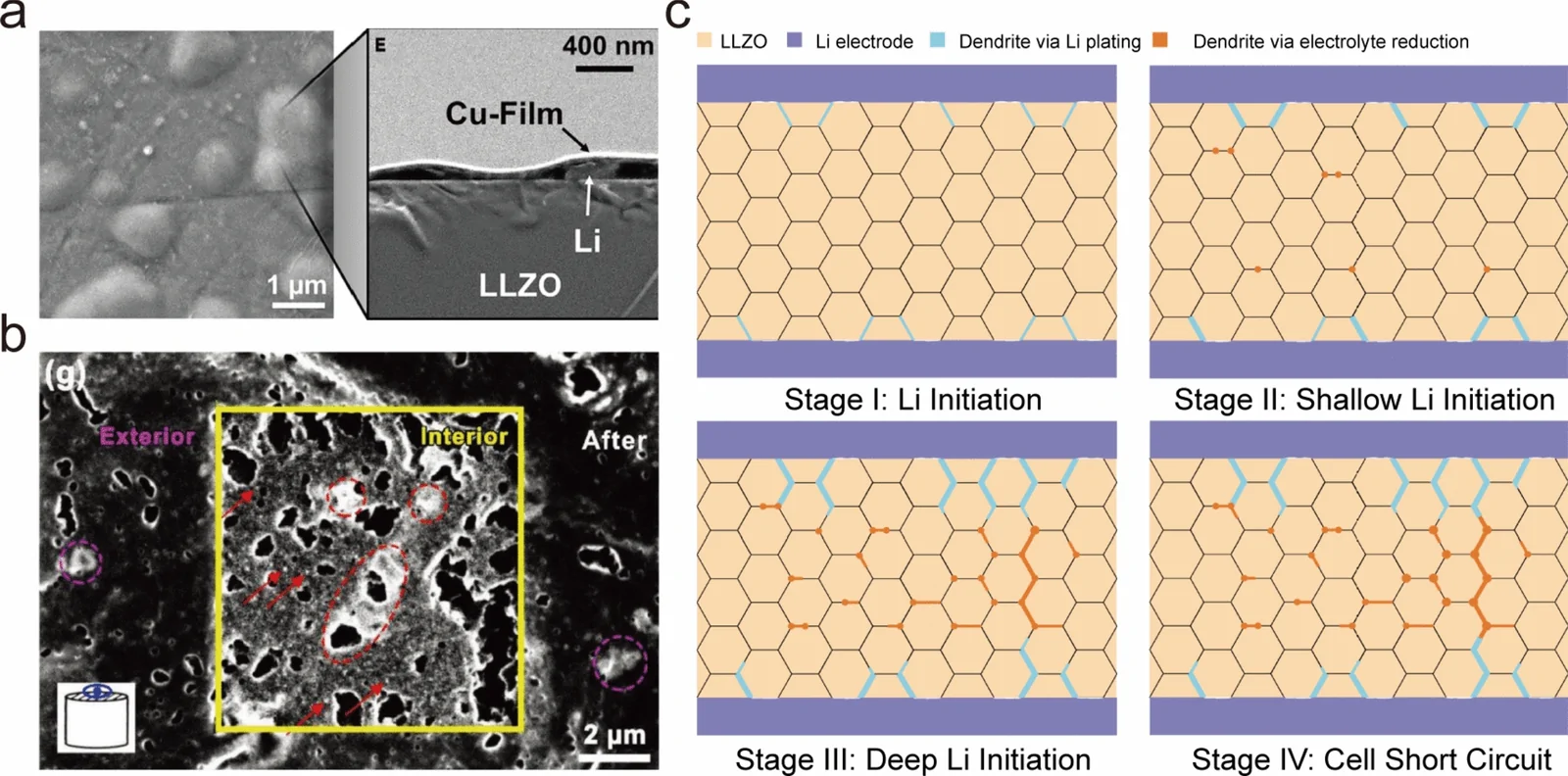
**a** Nucleation at interface in LLZO (Reproduced with permission from Ref. [28], Copyright 2019, Elsevier). **b** Nucleation at interface in grain boundary (Reproduced with permission from Ref. [57], Copyright 2019, Wiley–VCH). **c** Four stages of dendrite nucleation in LLZO (Reproduced with permission from Ref. [58], Copyright 2025, Springer Nature)

In solid-state batteries, lithium nucleation is understood to occur at two primary locations: the anode–electrolyte interface and internal defects within the SSE, such as pores or grain boundaries [56]. Krauskopf et al. [28] provided evidence for interfacial nucleation by observing non-uniform lithium deposition on the surface of LLZO electrolytes (Fig. 6a). By contrast, Mo et al. [57] observed that lithium metal deposits inside a LiBH_4_ electrolyte, supporting the possibility of internal nucleation (Fig. 6b). Gu et al. [56] proposed that interfacial nucleation generally dominates because it offers the lowest electronic resistance.

However, internal nucleation can also occur if electronic conductivity is present within the grain boundaries and pores of the electrolyte [55]. More recently, Liu et al. [58] demonstrated that these are not mutually exclusive modes but rather parts of a dynamic and coupled process. Using both NMR and MRI, they described a four-stage failure pathway in LLZO (Fig. 6c): (1) initial inhomogeneous nucleation at the interface; (2) a deposition pause accompanied by an amorphous to crystalline phase transition; (3) subsequent deposition within the grain boundaries of the electrolyte; and (4) connection of interfacial and internal deposits, leading to short-circuiting. Overall, Wang et al. [59] provided a unifying concept that categorizes all nucleation sites as defect regions within the solid-state battery (SSB), including grain boundaries, pores, impurities, and interfacial imperfections such as gaps or cracks [53].

**Fig. 7:**
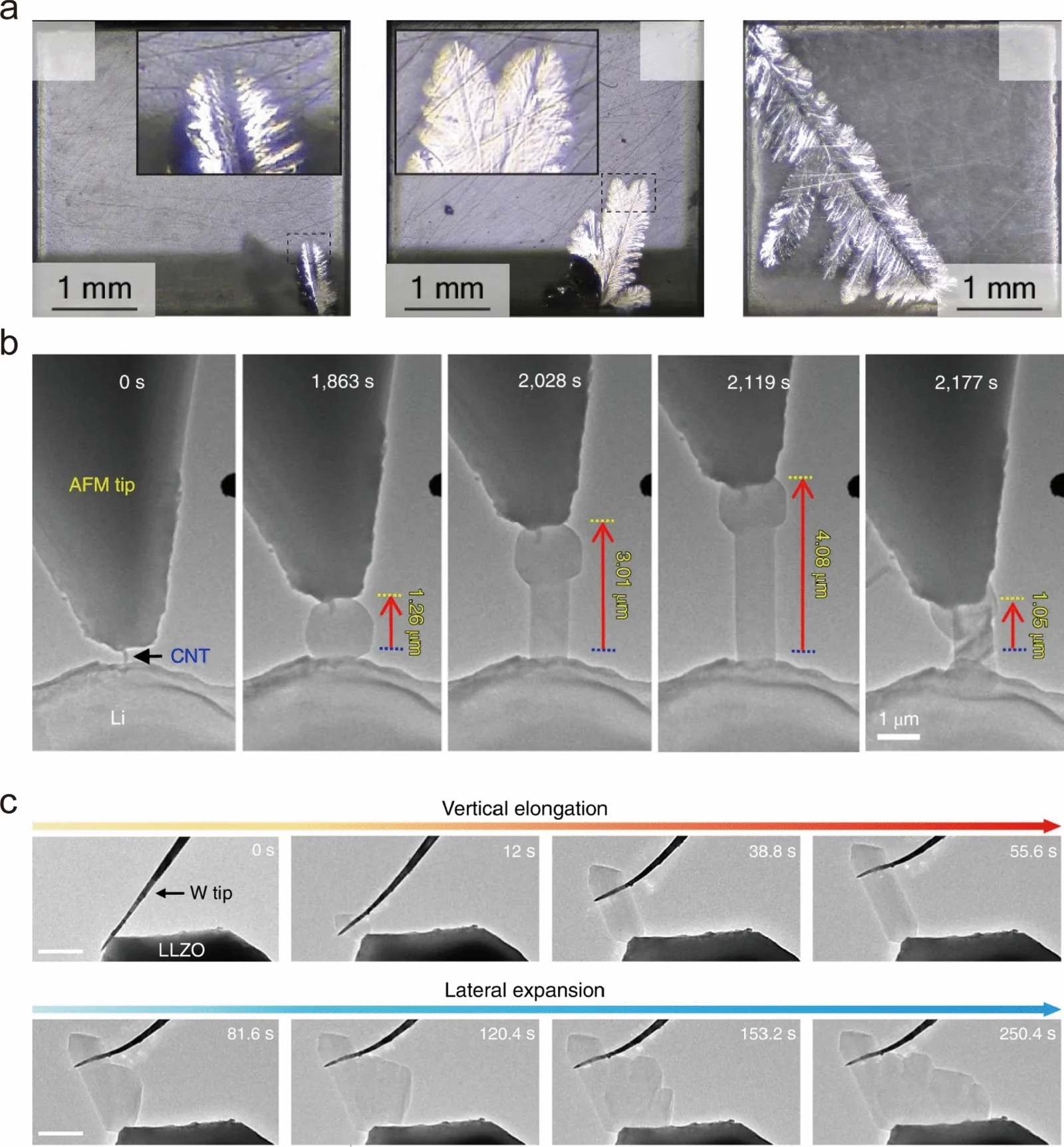
**a** Three morphologies induced by dislocations in LLZTO (Reproduced with permission from Ref. [61], Copyright 2024, Springer Nature). **b** Transition from spherical to whisker morphology (Reproduced with permission from Ref. [62], Copyright 2020, Springer Nature). **c** Dendrite growth shifts from longitudinal to lateral (Reproduced with permission from Ref. [63], Copyright 2022, Springer Nature)

The morphology and growth dynamics of dendrites within SSEs are highly diverse and are governed by local stresses, defects, and material properties. Using in situ video microscopy, Kazyak et al. [60] identified four distinct morphologies in LLZO: linear (unidirectional), branched (tree-like), delaminating (related to electrolyte fracture), and diffuse (spreading along grain boundaries). Dislocations have also been identified as key factors influencing dendrite propagation. As shown in Fig. 7a, dislocations in LLZTO have been demonstrated to facilitate three growth modes [61]: leaf-like branching along fracture pathways, single-path growth guided by dislocation density, and edge growth at lattice defects. It is worth noting that growth mechanisms are not fixed. Combining in situ ETEM and AFM, Zhang et al. [62] observed a morphological transition in a Li_2_CO_3_ layer (Fig. 7b). Initially, diffusion-controlled deposition created spherical particles. Upon reaching a critical internal pressure, a fracture-induced root-growth mechanism was activated, triggering a morphological transition from spherical to whisker-like. Similarly, Gao et al. [63] used a metal probe to create a localized hot spot on LLZO and observed via TEM that lithium initially followed a root-growth mode at this high-flux point (Fig. 7c). However, as deposition continued, the growth mode shifted from longitudinal to lateral expansion, with the lateral growth rate eventually exceeding the longitudinal rate.

#### Sodium Metal Anode System

**Fig. 8:**
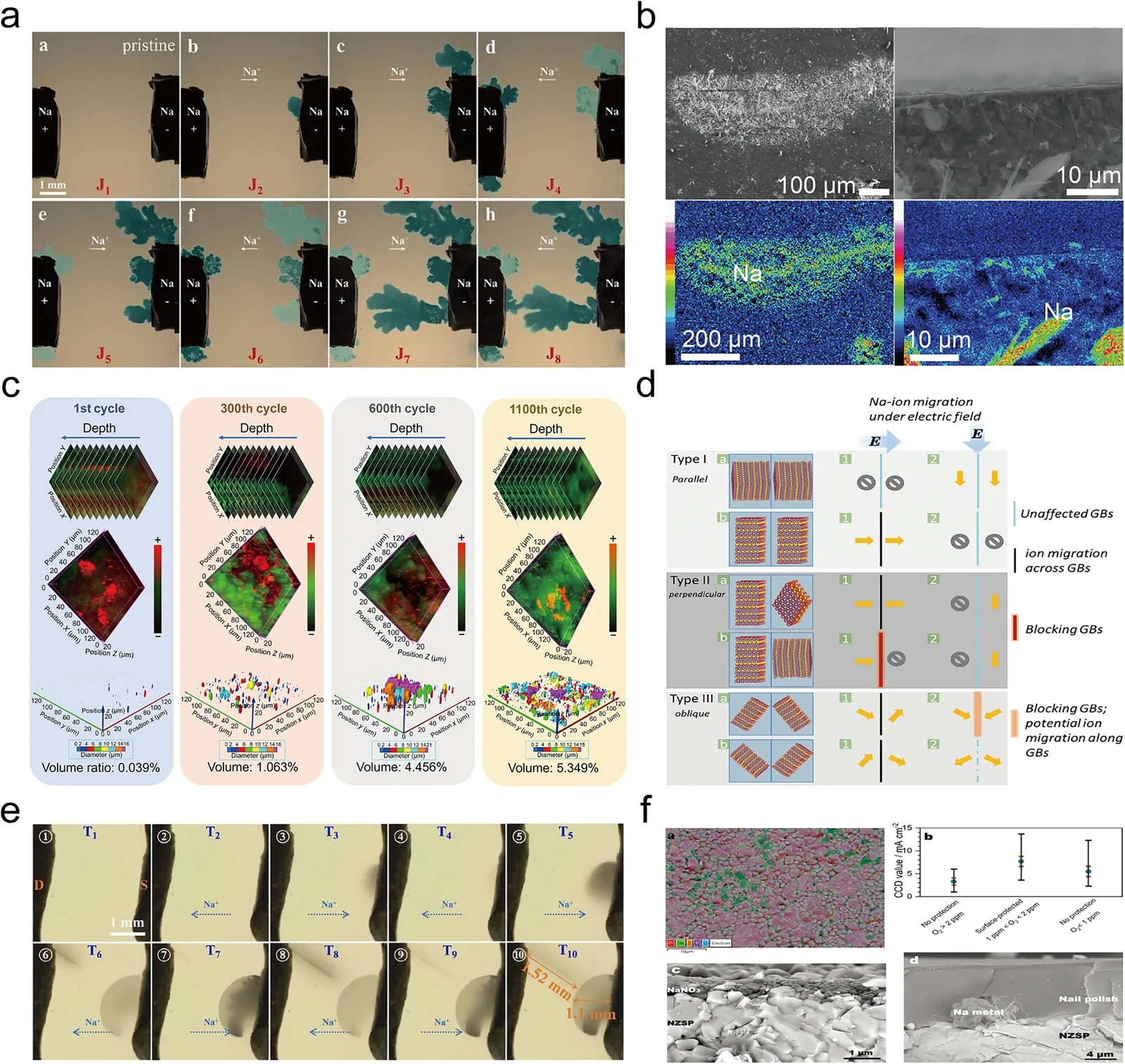
**a** Surface (Reproduced with permission from Ref. [65], Copyright 2023, Royal Society of Chemistry) and **b** grain boundary (Reproduced with permission from Ref. [66], Copyright 2024, Wiley–VCH) growth of sodium dendrite. **c** Fluorescence display of sodium dendrite evolution in NZSP (Reproduced with permission from Ref. [67], Copyright 2024, American Association for the Advancement of Science). **d** Three types of grain boundary (Reproduced with permission from Ref. [68], Copyright 2023, Wiley–VCH). **e** Interactive evolution of dendrites and cracks (Reproduced with permission from Ref. [71], Copyright 2025, Wiley–VCH). **f** Sodium deposition at the three-phase interface (Reproduced with permission from Ref. [72], Copyright 2022, Wiley–VCH)

Similar to lithium deposition, sodium deposition in SSEs occurs mainly at the anode–electrolyte interface and within internal grain boundary regions. Sun et al. [64] used synchrotron X-ray tomography to identify a continuous, electrochemically inert deposition layer at the interface between the anode and Na_4_(B_12_H_12_)(B_10_H_10_), which grew thicker during cycling. As shown in Fig. 8a, in situ optical observation of a Na-*β*″-Al_2_O_3_ electrolyte revealed that dendrites consistently nucleated and grew from the anode surface [65], even as the current density increased from 0.1 to 0.8 mA cm^−2^. By contrast, substantial evidence for internal nucleation has been observed in NASICON-type electrolytes. Gao et al. [66] used SEM and EDS to detect preferential sodium deposition along grain boundaries and pores within an NZSP electrolyte, forming needle-like or sheet-like structures (Fig. 8b). According to their analysis, the primary mechanism of internal nucleation is the local accumulation of Na^+^ at grain boundaries, where ionic conductivity is typically lower than in the bulk. This creates a concentration gradient that drives the sodium reduction reaction.

Using fluorescence tomography of Eu^3+^-doped NZSP, Yang et al. [67] visualized this process, observing the initial deposition of isolated sodium islands at the grain boundaries, which then merged into a continuous dendrite network over repeated cycles (Fig. 8c). Providing a more detailed crystallographic explanation, Ding et al. [68] proposed that the relative orientation of conduction planes between adjacent grains governs Na^+^ accumulation. They classified grain boundaries into three types and found that nucleation specifically occurs at Type III b2 boundaries, where oblique conduction planes form a bottleneck for ion transport (Fig. 8d). A variety of factors influence the morphology of sodium deposits. Ortmann et al. [69] discovered that in an NZSP system, a low current density (100 μA cm^−2^) resulted in island-like or whisker-like deposits, whereas a high current density (1000 μA cm^−2^) produced a uniform, dense layer morphology. Liu et al. [70], using an ETEM-AFM platform, observed various nanoscale shapes, including rods, spheres, and cubes, in a Na_2_CO_3_ model electrolyte. They found that the growth orientation was influenced by voltage, with higher voltages promoting growth along specific crystallographic directions.

Furthermore, cracks play a critical role in dendrite development. Wang et al. [71] used in situ observation to monitor the simultaneous growth of dendrites and cracks in NZSP. As shown in Fig. 8e, during the initial stage of the cycling phase, sodium was deposited or stripped through diverging cracks on the stripping side (S-side). Later, dendrites rapidly grew along cracks on the deposition side (D-side). Short-circuiting eventually occurred when dendrites from both sides connected. This process demonstrates a self-reinforcing cycle in which dendrite growth accelerates crack propagation, and cracks, in turn, create new sites for further dendrite formation. Beyond these factors, Ma et al. [72] highlighted the importance of the atmosphere, proposing that dendrite initiation in NZSP occurs at a three-phase boundary involving sodium, the SSE, and the surrounding atmosphere (Fig. 8f). They demonstrated that the critical current density (CCD) could reach 5.5 mA cm^−2^ in a low-oxygen environment, whereas it was much lower in atmospheres with high oxygen or water content. They compared the effects of nail polish and NaNO_3_/NaCl coatings on atmospheric exposure-induced changes in the critical current density. Dendrite growth remained visible on the surface beneath the nail polish coating, while the NaNO_3_/NaCl coating effectively prevented it. The is likely because the nail polish cannot fully block localized atmospheric penetration, indicating that dendrites not only grow along grain boundaries but also propagate across the grain boundary surfaces exposed to air.

The preceding sections have outlined the main experimental factors that influence dendrite development, such as substrate properties, current density, reaction kinetics, and material defects. These varied empirical observations can be unified and understood as macroscopic effects of interactions among underlying physical fields. All battery systems involve the electric field, ion concentration gradients, and interfacial chemical reactions. In solid-state batteries, however, a crucial interaction emerges with the stress field, which governs the combined growth of dendrites and cracks. The diverse morphologies and growth behaviors observed in experiments are thus primarily driven by the intricate coupling among these physical fields. The following sections explore how dendrite formation occurs from this multiphysics perspective, starting with the effects of each physical field individually and then examining their coupled interactions. A summary of the key experimental findings is provided in Table 1.

**Table 1:** Comparison of dendrite evolution behaviors in different electrolyte systems

System	Electrolyte	Conditions (mA cm^−2^)	Mechanism	Morphology	References
Liquid Li	LiPF_6_/EC:DEC = 1:1	0.2, 1.0, 5.0	Deposition rate dominance to the mass transfer limitation	Spherical columnar porous	[30]
	LiPF_6_/EC:DMC = 1:1	1 ~ 50	*V*_dep_ < *V*_SEI_, Root growth	Whisker-like	[40]
			V_dep_ > V_SEI_, Tip growth		[41]
			(LiPF_6_/Cu) Heterogeneous nucleation	(LiPF_6_/Cu) Dense columnar	[45]
	LiFSI, LiPF_6_ (DOL/DME, LiNO_3_)	5	(LiFSI) Homogeneous nucleation	(LiFSI) Mossy	
LiquidNa	NaPF_6_ (DME)	0.2–5	Interface chemical reaction	Micro-scale particle aggregation	[31]
	NaTFSI (PC)	0.5, 1.0, 2.0	Progressive, Instantaneous	Dense deposition, porous structure	[47]
	NaAlCl_4_·2SO_2_	0.3–1.5	Surface growth	Cubic morphology	[51]
Solid	LLZO, LLZTO	0.2	electrolyte fracture and grain boundary	Linear branched layered diffusive	[61]
Li	Li_2_CO_3_	10–30	Fracture-induced root growth	Spherical and whisker-like	[63]
	LLZO	− 0.5, − 1.0, − 5.0	Interface nucleation, Grain interior deposition	Interface spots and grain interior network	[71]
Solid	NZSP	≥ 0.3	Na^+^ accumulates at grain boundaries	Needle/sheet-like along grain boundaries	[66]
Na	NZSP	100 ~ 1000	Influence of current density on filling density	(Low current) Island/Whisker-like	
				(High current) Dense layered	[70]
	Na-*β*″-Al_2_O_3_	0.1–0.8	Growth at the anode-electrolyte interface	Surface dendrites	[72]
	NZSP	1	Atmospheric components induce dendrite initiation	Expand outward along the surface	[73]

## Dendrite Growth Mechanisms in Solid-State Li/Na Batteries: A Multiphysics Perspective

Mass transfer and electrochemical reactions in secondary batteries are driven by interconnected electric, chemical, and ion concentration fields. The temperature field, governed by principles such as the Arrhenius equation, significantly affects the kinetics of interfacial processes and reaction rates. In solid-state batteries, the stress field is equally crucial, as it can modify interfacial contact and ion transport, potentially leading to preferential dendrite growth and crack propagation. Therefore, the nucleation, growth, and evolution of dendrites are determined by the coupled interplay of the electrochemical, thermal, and mechanical fields.

Given the difficulty of isolating the electric, chemical, and ion-concentration fields within an electrochemical system, they are collectively referred to as the "electrochemical field" for the purposes of this review. To systematically examine the principles underlying dendritic evolution, this chapter describes how each physical field influences dendrite growth. This analysis is supported by relevant physical models, including thermodynamic, space charge, stress and plastic deformation, thin-film growth, and phase-field dynamics model [73, 74]. These models describe dendrite nucleation, morphological evolution, and failure modes from the perspective of different physical mechanisms.

However, during the actual operation of a battery, various physical fields do not exist in isolation. Instead, they interact with each other through material parameters, interface states, and boundary conditions, forming a complex coupled system. Based on the differences in coupling strength and feedback mechanisms, multi-physical field coupling can generally be divided into two categories: weak coupling and strong coupling. In the weak coupling framework, the interactions between physical fields are mainly unidirectional or sequential. For example, temperature changes indirectly affect deposition behavior by modifying electrochemical kinetic parameters, but there is no explicit bidirectional feedback between field variables. In the strong coupling framework, electrochemical, thermal, and mechanical field variables are directly coupled within the governing equations. A small disturbance in any physical quantity may be amplified through feedback mechanisms and reshaping the interface evolution path. For instance, the stress field modifies the interface reaction barrier, while the deposition morphology in turn reconstructs the stress distribution and local heat generation.

Building on this framework, this chapter will discuss the electrochemical, thermal, and stress fields, their interactions, and the contrasting influences of weak versus strong coupling feedback on dendrite stability. It should be noted that current research on the physical field of solid-state sodium battery systems remains still relatively limited. Therefore, this chapter will focus on the research of physical field models of solid-state lithium batteries, while introducing relevant findings from solid-state sodium battery systems at key points and highlighting the distinct research characteristics arising from differences in their physicochemical properties. Furthermore, to enable fundamental comparison and provide a theoretical foundation, this chapter necessarily refers to relevant models developed for liquid battery systems, which serve as an essential basis for understanding the dendrite behavior in solid-state systems.

### Single Physics Field

#### Electrochemical Field

**Fig. 9:**
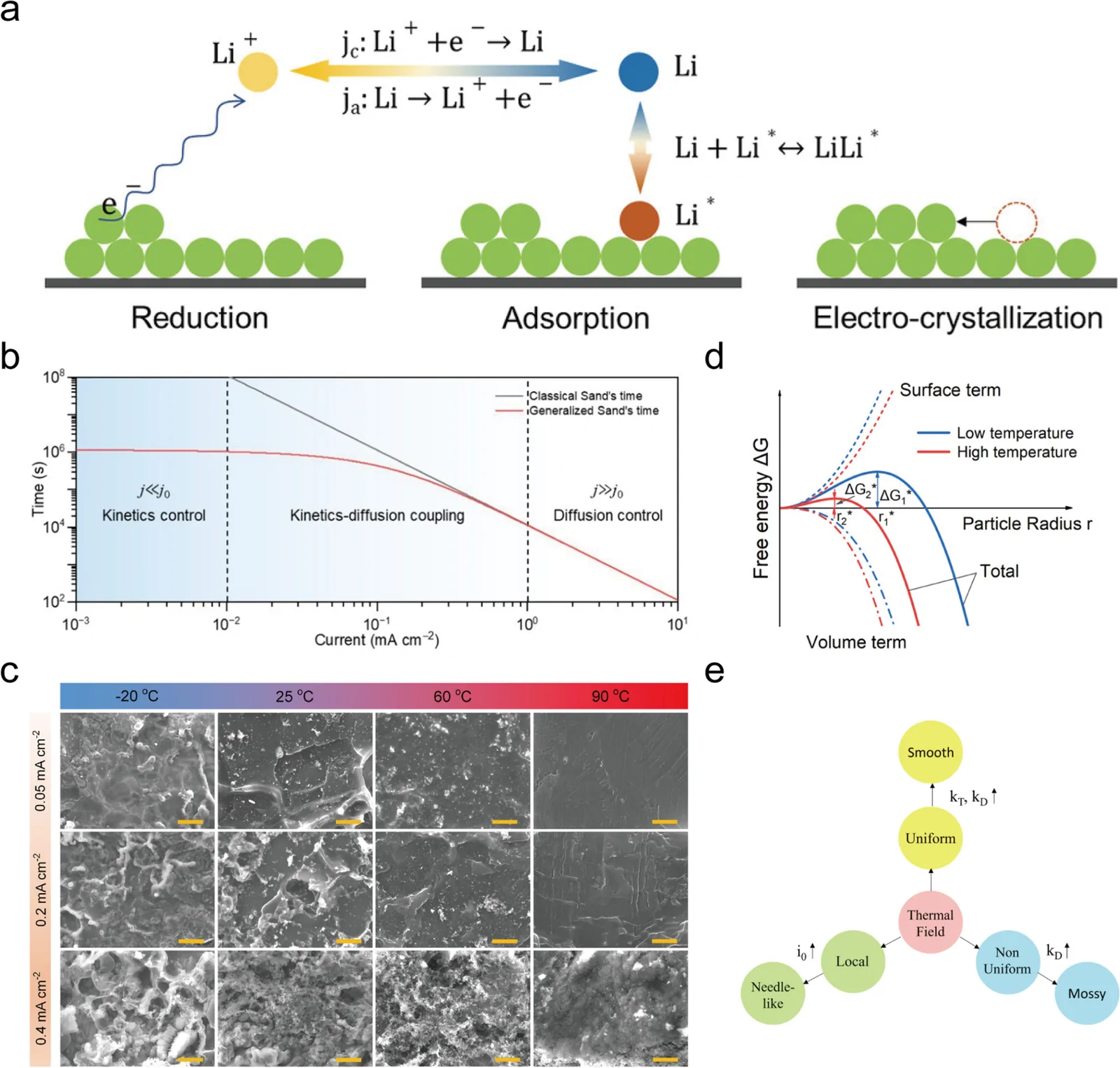
**a** Schematic diagram of lithium electrochemical deposition (Reproduced with permission from Ref. [75], Copyright 2021, Wiley–VCH). **b** Time–current curve based on Sand’s Time model (Reproduced with permission from Ref. [78], Copyright 2025, American Chemical Society). **c** Lithium deposition morphology under different current density and temperature conditions and **d** free energy-radius curves at different temperature (Reproduced with permission from Ref. [79], Copyright 2022, Elsevier). **e** Dendrite morphology under different temperature (Reproduced with permission from Ref. [80], Copyright 2020, American Chemical Society)

As illustrated in Fig. 9a, dendrite formation is an electrocrystallization process wherein metal cations are reduced to metal atoms, which then adsorb onto the electrode surface [75]. This process is influenced by the electrochemical field, including current density, potential distribution, and ion concentration gradients.

A foundational model for predicting dendrite initiation time from an electrochemical perspective is the Sand’s Time model [76]. According to this model, dendrites form when the Li^+^ diffusion rate is insufficient to meet the demand of the applied constant current, causing the ion concentration at the interface to drop to zero. The moment at which the interface concentration reaches zero under a constant current is termed Sand’s Time, and the corresponding current density is defined as the CCD [77]. This condition indicates that ion migration driven by the electric field overtakes diffusion driven by concentration gradients, thereby resulting in dendrite formation. However, the Sand’s Time model is less accurate at low currents because it does not account for electrochemical kinetics. To address this limitation, Yang [78] proposed a modified Sand’s Time model that includes a correction factor to account for the interaction between interfacial reaction kinetics and mass transfer. As shown in Fig. 9b, this revised model indicates that dendrite growth is dominated by kinetics at low current densities and by diffusion at high current densities, with a nonlinear relationship between current density and dendrite initiation time in the intermediate range. This modification enables a consistent description of dendrite behavior across various current regimes.

It should be emphasized that both the original Sand’s Time model and its modified version were primarily developed for liquid electrolyte systems and general electrocrystallization scenarios and therefore require specific modifications when applied to SSB systems, which exhibit unique ion transport mechanisms and interface properties distinct from liquid systems. The key considerations for adapting these models to SSBs are summarized as follows.

1. Ion transport mechanisms. Ion transport in SSEs differs fundamentally from that in liquid electrolytes. Solid electrolytes generally exhibit lower ionic conductivity, and ion diffusion is often restricted by grain boundaries, lattice defects, and interface contact conditions. A key distinction lies in the ion transport number. The solid electrolytes typically have a transport number of unity, whereas liquid electrolytes exhibit values of 0.2–0.3 due to the presence of both cationic and anionic charge carriers. Consequently, the Sand’s Time model for liquid systems is established based on the coupled transport dynamics of both cations and anions, whereas the model for solid electrolytes must account for the migration of cations and electrons. This difference in transport number further leads to distinct thermodynamics of dendrite formation between the two systems. In liquid electrolytes, the concentration gradient near the electrode–electrolyte interface is strongly governed by the dynamic motion of anions in addition to that of cations. In solid electrolytes, by contrast, the absence of mobile anions means the interfacial concentration gradient is determined primarily by cation transport and electron dynamics. Therefore, the diffusion coefficient used in the Sand’s Time model cannot be directly adopted from liquid systems; instead, it needs to be modified to reflect the actual ion transport efficiency in solid electrolytes, accounting for factors such as bulk resistance and grain boundary impedance.
2. Interface characteristics. The solid-state electrode–electrolyte interface is a solid–solid contact interface, exhibiting higher contact resistance and more complex interfacial reactions than the liquid–solid interface in conventional batteries. The interfacial charge transfer resistance and ion migration resistance at the solid–solid interface will significantly influence the interfacial ion concentration distribution and should therefore be incorporated into the model as additional correction terms to improve the accuracy of dendrite initiation time prediction.
3. SEI layer and mechanical contact. The formation and stability of the SEI layer at the solid-state interface, as well as the interfacial mechanical contact state (e.g., contact gaps caused by volume changes), further alter ion transport paths and interfacial reaction kinetics. These factors need to be considered in the model modification to avoid discrepancies between theoretical predictions and actual dendrite growth behavior in solid-state batteries.

In summary, the adaptation of the Sand’s Time model to SSB systems requires targeted corrections based on the unique characteristics of solid electrolytes and solid–solid interfaces. Such adaptations are essential for establishing a more accurate theoretical foundation for dendrite prediction and mitigation in solid-state batteries.

#### Thermal Field

Research has shown that high temperatures promote uniform lithium deposition in liquid battery systems, while low temperatures encourage lithium dendrite growth [81, 82]. This temperature-dependent behavior is also observed in SSB systems. Sharafi et al. [83] reported that dendrite behavior at the Li/LLZO interface is highly dependent on temperature, with dendrite growth being notably suppressed as the temperature increased from 30 to 160 °C. They attributed this to the increased fluidity of lithium metal at higher temperatures, which fills interfacial flaws, as well as to a reduction in the interface charge-transfer resistance.

Similarly, Luo et al. [79] observed the evolution of lithium dendrites over a temperature range of – 20–90 °C. As shown in Fig. 9c, at low temperatures (− 20 °C), lithium deposition creates a honeycomb-like porous structure, with dendrites growing vertically within the pores of the solid-state electrolyte. Conversely, at high temperatures (90 °C), a dense, lateral deposition layer forms, characterized by a much larger nucleation size and a significantly lower nucleation density. Furthermore, molecular dynamics simulations (Fig. 9d) revealed that increasing the temperature decreases the critical nucleation radius and surface energy of lithium metal, thereby lowering the free energy barrier and promoting lateral deposition. These findings, however, assume a uniform temperature distribution.

Furthermore, Vishnugopi et al. [80] integrated the Biot number into a coarse-grained kinetic Monte Carlo model to systematically explore how temperature uniformity affects lithium dendrite formation. The model considered three scenarios: a uniform temperature field, a non-uniform temperature field, and localized hotspots. The results showed that, under a uniform temperature field, higher temperatures enhance lithium-ion self-diffusion, which promotes uniform lithium deposition. In contrast, localized hotspots increase the exchange current density, leading to the preferential growth of dendrites. In these regions, higher temperatures increase the local exchange current density, resulting in the formation of needle-like dendrites. As shown in Fig. 9e, the influence of temperature on lithium deposition morphology under different temperature distributions is primarily governed by its effect on three key parameters: the charge transfer exchange current density (*i*_0_), the self-diffusion coefficient of lithium ions (Kᴅ), and the diffusion rate of lithium ions in the electrolyte (Kᴛ).

#### Stress Field

**Fig. 10:**
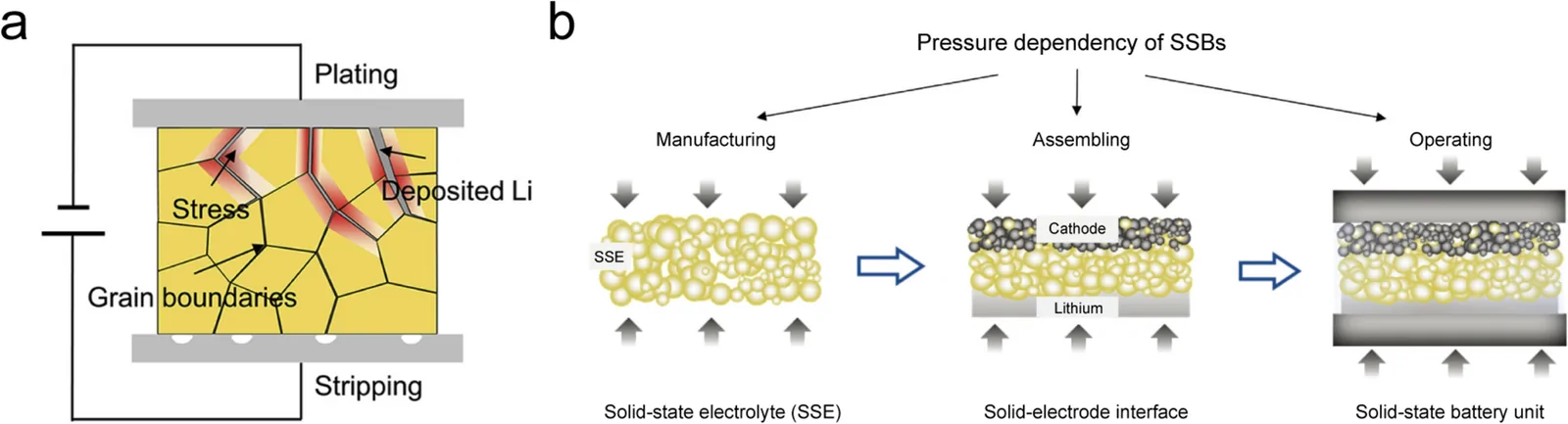
**a** Diagram of internal (Reproduced with permission from Ref. [87], Copyright 2022, Elsevier) and **b** external stress (Reproduced with permission from Ref. [86], Copyright 2024, Wiley–VCH)

Stress plays a critical role in the formation and growth of dendrites in solid-state batteries. Yildirim et al. [61] found that dislocation structures induced by stress at the dendrite tip lead to branching of lithium dendrites within solid electrolytes. Furthermore, a fracture mechanics model revealed that external stresses can change the direction of metal dendrites [84], indicating that stress affects dendrite growth pathways. Stress at the metal anode/solid-state electrolyte interface can be classified as either internal or external. Internal stresses [85], arising from uneven lithium-ion deposition and the volumetric expansion of lithium metal, mainly result from phenomena at the solid–solid interface and within the electrolyte (Fig. 10a). External stresses, by contrast, arise from sources such as stack pressure during battery assembly and operation, as well as manufacturing pressures [86] (Fig. 10b).

Early research on the effects of stress focused on the shear modulus of SSEs. The linear-elastic model proposed by Monroe and Newman suggested that lithium dendrite growth could be suppressed if the shear modulus of the polymer solid-state electrolyte were more than twice that of lithium metal [88]. However, Barai et al. [89] later demonstrated that this condition is only effective when the lithium metal is under existing stress and at low current density. Their results also showed that dendrite growth is prevented in initially stress-free lithium, even with a low electrolyte modulus. Moreover, increasing the polymer electrolyte modulus to 20 times that of lithium metal enables stable deposition at up to 75% of the limiting current density [90]. These results highlight the significant impact of the mechanical state of lithium metal on dendrite growth.

**Fig. 11:**
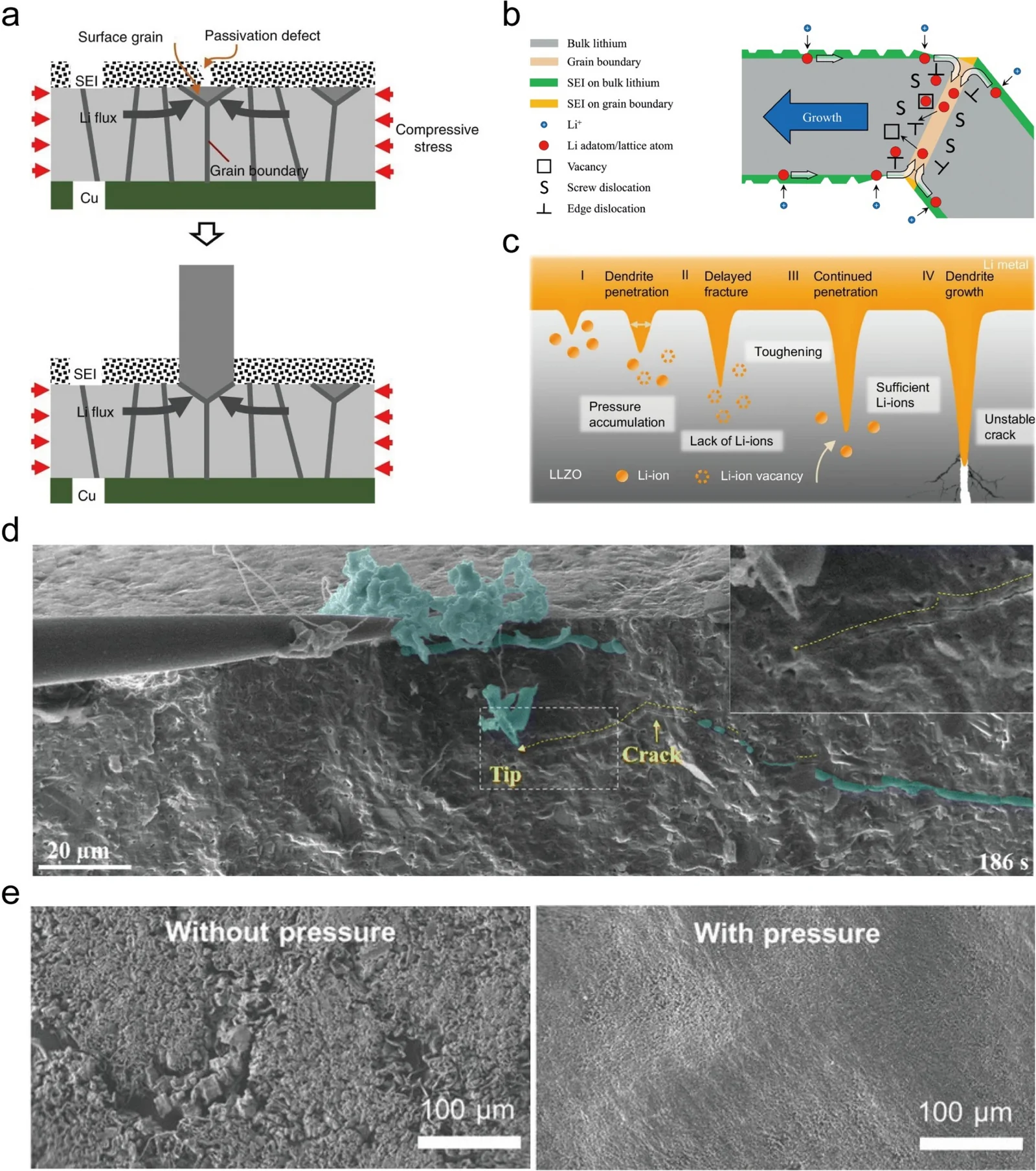
**a** Schematic diagram of stress-driven model (Reproduced with permission from Ref. [91], Copyright 2018, Springer Nature). **b** Vacancy filling and dislocation creep mechanism (Reproduced with permission from Ref. [92], Copyright 2022, Royal Society of Chemistry) and **c** four stages of dendrite growth (Reproduced with permission from Ref. [93], Copyright 2025, Springer Nature). **d** Sodium dendrite growth and crack propagation (Reproduced with permission from Ref. [65], Copyright 2023, Royal Society of Chemistry). **e** Lithium deposition morphology with and without pressure (Reproduced with permission from Ref. [94], Copyright 2021, Wiley–VCH)

Internal stress is widely recognized as a primary factor in lithium dendrite formation. For example, Wang et al. [91] examined lithium deposition on rigid copper foil and soft substrates, observing sharp dendrites on the stiff substrate and more uniform deposition on the flexible substrate, which formed wrinkles to relieve stress. Based on these observations, they proposed a stress-driven model wherein compressive stress arises from the insertion of atoms into grain boundaries during non-equilibrium deposition. This stress causes lithium atoms to diffuse along these boundaries to the surface, where they escape through defects in the SEI via creep, leading to whisker-like dendrites growing in a root-like manner (Fig. 11a). Considering the significant difference in modulus between sodium and lithium, Na is more susceptible to creep deformation. Therefore, the stress-driven mechanism of dendrite growth exhibits a much greater influence in sodium metal battery systems.

From another perspective, Becherer et al. [92] suggested that stress induces plastic deformation in lithium, resulting in defects that facilitate dendrite growth via vacancy filling and dislocation climb (Fig. 11b). In single-crystal electrolytes, dendrite growth is associated with electrolyte fracture. Zhang et al. [93] used molecular dynamics simulations to reveal that continuous lithium deposition causes internal stress accumulation, which eventually leads to fracture of the LLZO solid electrolyte at the dendrite tip. They outlined four stages in this process (Fig. 11c): (1) lithium initially deposits at defects, leading to stress buildup; (2) dendrite growth pauses as the stress approaches the fracture toughness of the electrolyte; (3) once this threshold is exceeded, a crack propagates from the tip; and (4) the dendrite extends along the newly formed crack. As shown in Fig. 11d, Geng et al. [65] used in situ scanning electron microscopy to observe that sodium deposition in Na-*β*″-Al_2_O_3_ leads to a gradual stress buildup. This stress causes electrolyte fractures that form pathways for subsequent sodium deposition, highlighting a “memory effect” in dendrite growth.

**Fig. 12:**
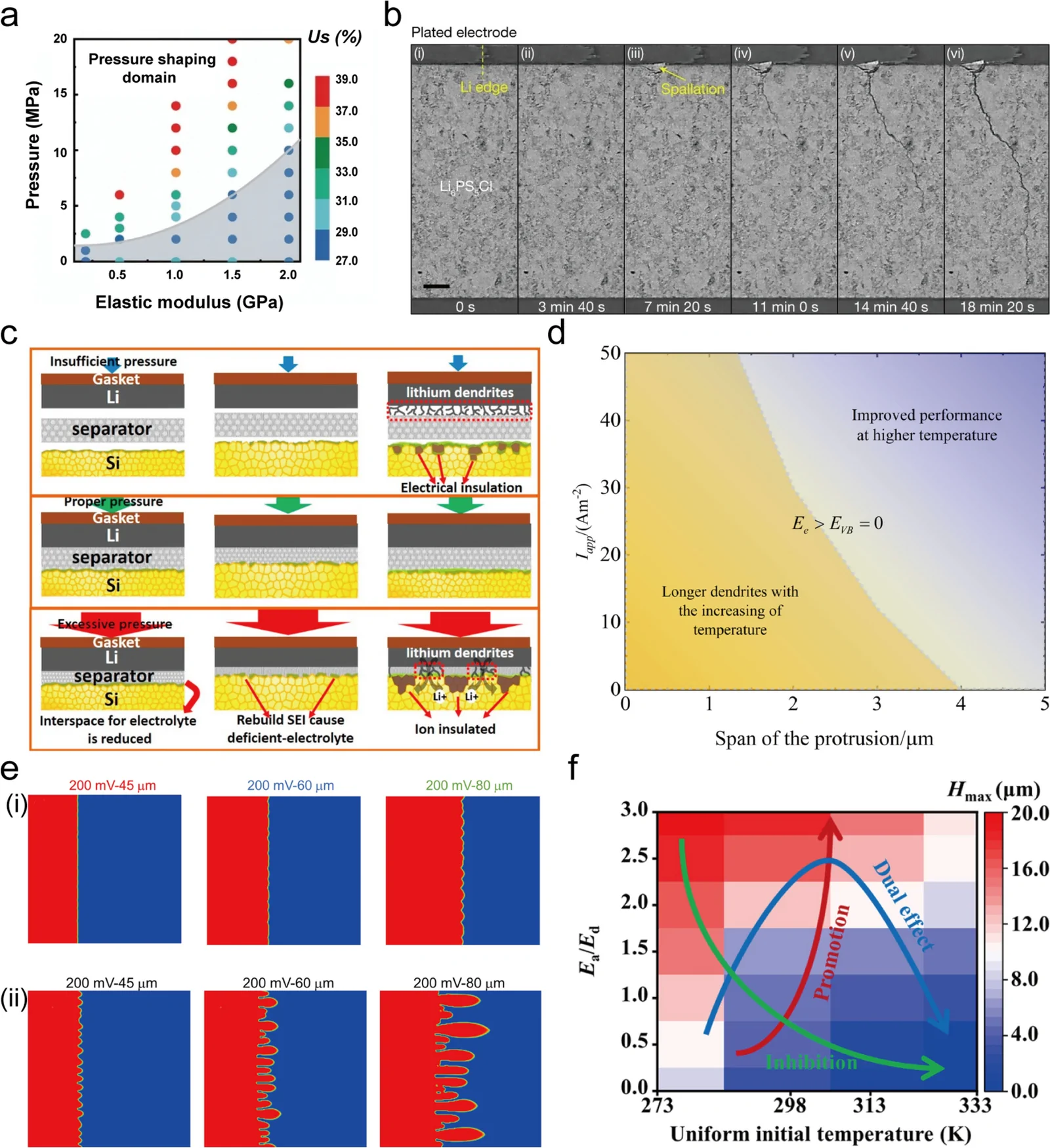
**a** Stress–strain modulus phase diagram (Reproduced with permission from Ref. [94], Copyright 2021, Wiley–VCH). **b** Dynamic evolution of crack in LPSC (Reproduced with permission from Ref. [18], Copyright 2023, Springer Nature). **c** Effect of dendrite suppression under different pressures (Reproduced with permission from Ref. [97], Copyright 2021, Elsevier). **d** Temperature, dendrite length, and activation energy phase diagram (Reproduced with permission from Ref. [99], Copyright 2023, Elsevier). **e** Dendrite length under different electrochemical energy barriers at high temperature (Reproduced with permission from Ref. [100], Copyright 2019, American Chemical Society). **f** The promoting, inhibiting, and dual effects of temperature on dendrite growth (Reproduced with permission from Ref. [101], Copyright 2023, American Association for the Advancement of Science)

External pressure also markedly affects the morphology of lithium deposition. Shen et al. [94] demonstrated that applied pressure can transform a loose, porous lithium deposit into a dense, smooth film (Fig. 11e). Using phase-field simulations, they constructed a pressure-electrolyte modulus phase diagram (Fig. 12a), revealing that, for a given electrolyte modulus, there is a critical stack pressure [95]. Dendrite growth is suppressed only when the external pressure exceeds this critical value [96]. This suppression arises from pressure-induced plastic deformation of the lithium metal.

However, some studies indicate that excessive external pressure can instead accelerate dendrite growth. Ning et al. [18] proposed that pressure can trigger a “wedge-expansion” mechanism, leading to premature cell failure. In their model, lithium initially penetrates subsurface microcracks in the solid-state electrolyte (Fig. 12b, Stages I–III). The applied external pressure then pushes the deposited lithium metal toward the crack tip, widening the fracture and encouraging further dendrite intrusion (Fig. 12b, Stages IV–VI). This stress-driven mechanism involving the coupled propagation of dendrites and cracks is also present in sodium metal battery systems [71]. These seemingly conflicting findings indicate that the effect of external pressure is highly dependent on its magnitude. As shown in Fig. 12c, insufficient pressure fails to prevent dendrite growth, while optimal pressure can suppress it [97]. Conversely, excessive pressure may cause lithium metal to creep into electrolyte cracks, exacerbating dendrite formation [98].

### Dual-Physical-Field Coupling

Although research on individual physical fields can reveal local mechanisms of dendrite growth, it neglects the essential coupling among electrochemical, thermal, and stress fields. To fully understand dendrite evolution, it is necessary to develop multiphysics models that account for the intricate interactions among these phenomena.

#### Electrochemical–Thermal Field Coupling

Although traditional electrochemical models indicate that high current density promotes dendrite formation, incorporating thermal effects reveals a more complex relationship. Li et al. [25] found that dendrites can undergo self-heating at high current densities, which can actually inhibit their growth. Through molecular dynamics simulations across a temperature range of 20–80 ℃, they observed that above 40 °C, the surface diffusion rate of lithium atoms sharply increases, leading to migration from dendrite tips into valleys. Consequently, the sharp dendrites fuse and smooth out after only 50 ps of annealing at 80 °C. These results demonstrate that electrochemical–thermal coupling can significantly alter dendrite behavior, leading to deviations from single-field prediction models.

Nevertheless, higher temperatures do not always suppress dendrite growth, as their effect depends on other kinetic factors, such as the development of the SEI. Cao et al. [99] described a competing process between SEI formation and electrochemical deposition. They characterized this competition by the relative activation energies of electrodeposition (*E*_VB_) and SEI formation (*E*_*e*_). As shown in Fig. 12d, high current densities and low surface curvatures, electrodeposition prevails, and higher temperatures promote uniform deposition by increasing Li^+^ diffusion. Conversely, at low current densities and high surface curvature, SEI formation dominates, and elevated temperatures can decrease the unevenness of the SEI, thereby accelerating dendrite growth.

Building on this duality, Hong et al. [100] used a nonlinear phase-field model incorporating energy conservation to investigate electrochemical–thermal coupling. Their results showed that the outcome depends on whether the electrochemical reaction barrier or the Li^+^ diffusion barrier dominates. When diffusion is the limiting factor, higher temperatures primarily enhance Li^+^ transport, leading to a more uniform ion flux and reduced dendrite formation (Fig. 12e(i)). By contrast, if the electrochemical reaction barrier limits the process, a high temperature accelerates the reaction, leading to rapid Li^+^ consumption at the interface and worsening dendrite growth (Fig. 12e(ii)). This effect is further intensified by a higher overpotential. The activation energy ratio associated with lithium-ion transport and reaction (*E*_a_/*E*_*d*_) determines whether temperature promotes or inhibits dendritic growth when the “rate” is substituted for the “barrier” [101]. As shown in Fig. 12f, the temperature increase will have dual effects on dendritic growth, promoting or suppressing it depending on the activation energy ratios.

#### Electrochemical–Stress Field Coupling

**Fig. 13:**
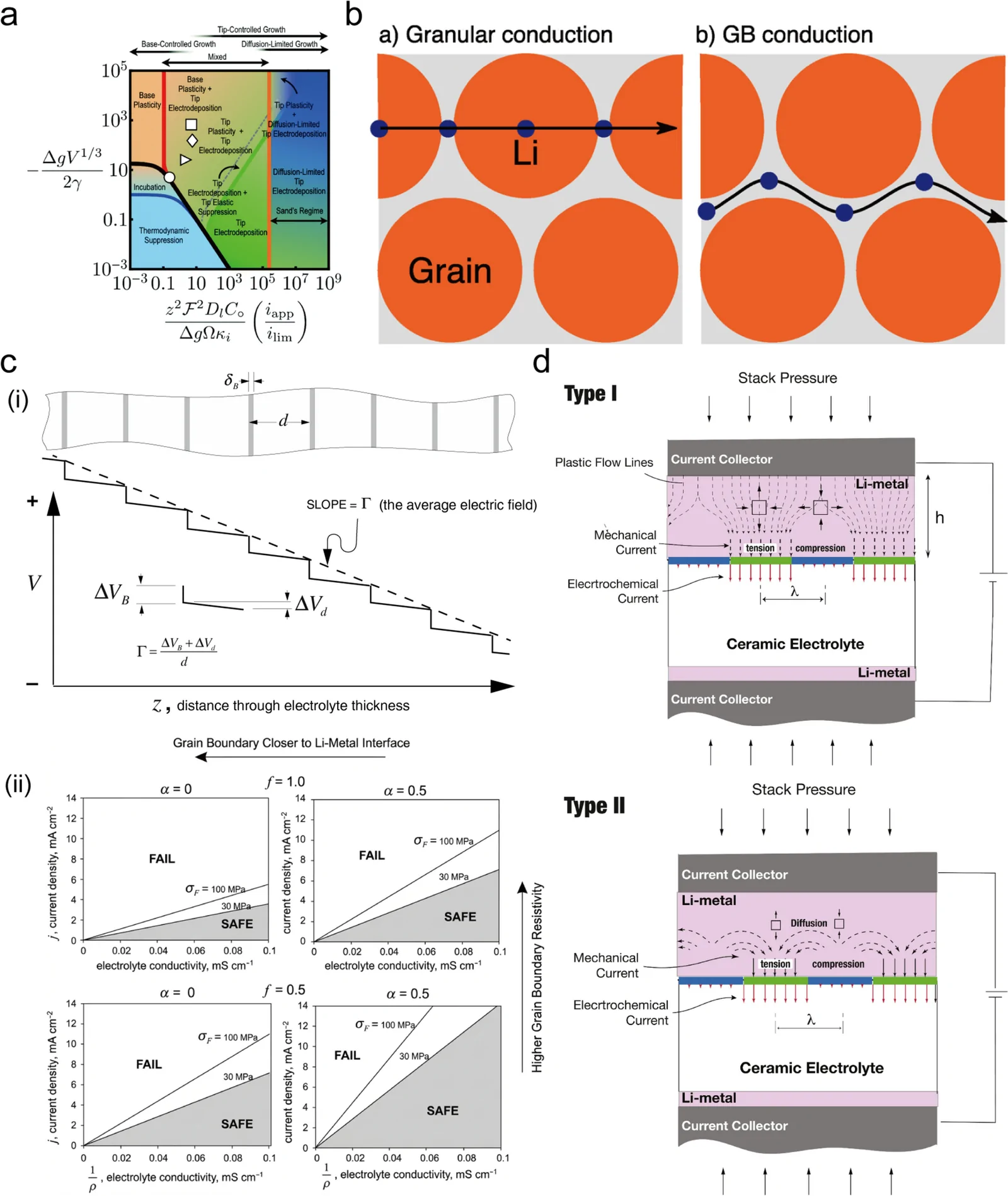
**a** Six patterns of dendrite growth (Reproduced with permission from Ref. [102], Copyright 2019, Royal Society of Chemistry). **b** Transgranular and intergranular growth (Reproduced with permission from Ref. [103], Copyright 2018, American Chemical Society). **c** (i) Grain boundary resistance model and (ii) current-limiting map (Reproduced with permission from Ref. [106], Copyright 2017, Elsevier). **d** Two mechanisms for mechanical current compensation of electrochemical current (Reproduced with permission from Ref. [110], Copyright 2021, Elsevier)

The electrochemical and stress fields are inherently strongly coupled. Integrating them reveals the complexity of lithium deposition. Based on extensive experimental observations, Jana et al. [102] proposed a model that classifies lithium growth into six distinct modes, primarily determined by current density. As shown in Fig. 13a lithium dendrite formation is entirely suppressed in the thermodynamic suppression zone (below the blue line) when the applied current density is below the critical threshold. As the current density increases, lithium nuclei form and gradually grow in the incubation zone (between the blue and black lines). At relatively low current densities, mechanical stress causes plastic flow of lithium, leading to the growth of columnar or mossy dendrites within the substrate-controlled zone (above the black line and to the left of the red line). At higher current densities, electrodeposition concentrates at the dendrite tips; however, the associated elastic strain energy locally inhibits vertical growth, resulting instead in lateral branching within the tip-controlled zone (to the left of the green line). The area between the substrate-controlled and tip-controlled zones is a mixed region where both plastic flow and tip-driven electrodeposition influence dendrite morphology. Beyond the orange line lies Sand’s zone, where dendrite growth is dominated by concentration gradient-driven deposition.

This model explains morphological diversity, but the physical pathways of dendrites in polycrystalline SSEs add further complexity, with growth occurring either between grains (intergranular) or through them (transgranular), as illustrated in Fig. 13b. Regarding sodium systems, Gao et al. [66] observed that sodium dendrites preferentially nucleate at grain boundaries of Na_3_Zr_2_Si_2_PO_12_ electrolytes and grow along them, attributing this behavior to the synergistic effect of local polarization potentials and stress accumulation at grain boundaries, based on theoretical analysis. The inferior mechanical stability and inherent defects at grain boundaries lead to severe stress concentration, which accelerates localized sodium deposition and establishes a self-reinforcing cycle, ultimately leading to dendrite penetration. Dawson et al. [103] established that the dendrite propagation pathway is influenced by the relative ionic resistances of the grain boundaries and the bulk crystalline grains. Transgranular growth occurs when the grain boundary resistance is much higher than the bulk resistance, whereas intergranular growth occurs when these resistances are comparable.

Bistri et al. [104] further clarified this distinction by developing a thermodynamically consistent damage model for LLZO, demonstrating that mechanical failure is the key step preceding dendrite growth. Their model suggests that stress acts as a thermodynamic driving force for electrodeposition, with tensile stress promoting deposition and compressive stress hindering it. Mechanical properties, therefore, govern the selection between intergranular and transgranular pathways. Grain boundaries are mechanically weaker due to their higher defect densities and lower fracture energy, making them prone to fracture under stress, thereby creating pathways for lithium deposition and leading to intergranular dendrite growth. By contrast, the crystalline grains possess superior mechanical properties. They can withstand higher stress levels before breaking, and when their fracture energy is finally surpassed, the dendrite grows directly through the grain in a transgranular manner. This illustrates a key concept: dendrite growth in polycrystalline electrolytes results from a balance between stress-induced fracture and electrochemical deposition.

A key approach to understanding electrochemical–mechanical coupling is to quantify how stress affects electrochemical parameters, such as the CCD and overpotential. For example, Hao et al. [105] modified Sand’s model by introducing a coupling coefficient, showing that stress in polymer electrolytes can increase the CCD and delay dendritic growth by enhancing ion transport. According to Raj and Wolfenstine [106], the compressive stress generated during lithium nucleation opposes the driving force from the local electrochemical potential. Moreover, the high resistance at grain boundaries increases the local electrochemical potential at the lithium anode/solid-state electrolyte interface. To quantify the combined effects of compressive stress and grain boundary resistance, they introduced an electrochemical–mechanical potential and proposed a grain boundary resistance model (Fig. 13c(i)). As shown in Fig. 13c(ii), they constructed a current-limiting map by including mechanical effects in the CCD equation. The model predicts that lithium dendrite formation is more likely when the grain boundary is closer to the anode (smaller α), when the grain boundary resistance is greater (larger f) and when the electrolyte has lower fracture toughness.

Additionally, Hu et al. [87] demonstrated that local tensile stress enhances deposition by reducing the nucleation energy barrier and making the overpotential more negative. Li and Monroe [107] provided a more detailed analysis of LLZO electrolytes by examining the frequency-dependent capacitive and resistive responses of the system to explore electrochemical and mechanical effects in the space-charge layer. Their model identifies two distinct regimes based on the characteristic frequency (*f*_0_). At low frequencies (*f* < *f*_0_), the system is dominated by resistive effects, leading to compression in the electrolyte bulk. At high frequencies (*f *> *f*_0_), capacitive effects occur, causing the bulk to be under tension relative to the interface. Importantly, they discovered that lithium nucleation occurs when a specific pressure drop is reached within the capacitive-dominated regime. Based on this, they established a direct link between critical pressure drop and the critical current density, and the interfacial compression increases the free energy barrier for lithium deposition at the interface relative to that at grain boundaries, thereby promoting lithium deposition within the grain boundaries and facilitating dendrite growth.

Barai et al. [108, 109] linked lithium deposition at LLZO grain boundaries to a kinetic overpotential driven by stress-induced current focusing. Their research indicated that this focusing is affected by both the ionic conductivity and Young’s modulus of the grain boundary, with ionic conductivity playing a more significant role. Meanwhile, Porz et al. [53] connected overpotential directly to stress, as minor overpotential levels can generate sufficient stress to initiate crack growth via the Griffith mechanism, thereby leading to dendrite formation. Building on these insights, to fully leverage the electrochemical–mechanical coupling effect for dendrite suppression in practical systems, several strategies can be employed, including but not limited to: (1) introducing interfacial compressive stress to reduce local electrochemical driving forces and raise the nucleation barrier; (2) improving the fracture toughness of electrolytes to suppress stress-induced crack initiation and propagation; (3) regulating grain boundary position, resistivity, and ionic conductivity to weaken current focusing and inhibit preferential deposition at grain boundaries; (4) controlling the operating overpotential to avoid crack extension driven by the Griffith mechanism, and (5) utilizing the resistance–capacitance response of electrolytes at different frequencies to avoid the critical pressure drop range that triggers metal nucleation.

External stress, such as stack pressure, can be examined within a similar framework, wherein mechanical forces generate an effective overpotential that facilitates mass transport. Raj [110] proposed two mechanisms by which mechanical current can offset non-uniform electrochemical current (Fig. 13d). In Type I, the stack pressure causes lithium creep from areas of high-to-low current density, perpendicular to the interface. In Type II, pressure promotes the lateral diffusion of lithium along the interface. Both mechanisms enhance mass transfer, leading to more uniform lithium deposition and thereby effectively increasing the critical current density. The Type II mechanism, in particular, exhibits a nearly linear relationship between the applied stack pressure and the critical current density.

It is important to note that the aforementioned electrochemical-stress coupling models and mechanistic insights are predominantly derived from studies on lithium metal systems. Consequently, when extending these modeling frameworks to sodium metal solid-state batteries, critical revisions to parameters, assumptions, and physical descriptors are required. Recent work by Singla et al. [111] systematically investigated the interfacial evolution behavior of sodium metal anodes during plating and stripping. Their results reveal that Na plating and stripping exhibit a pronounced kinetic asymmetry, attributed to the synergistic effects of inhomogeneous electrochemical reactions, ion transport polarization, interfacial stress concentration, and mechanical failure of the SEI. Mismatches among local current density, ion transport rate, and interfacial mechanical strength can trigger a sequential failure process including dendrite growth, SEI fracture, void formation during stripping, and dead sodium generation. Furthermore, stable and symmetric Na plating and stripping can only be achieved when the SEI exhibits sufficient toughness and uniformity, high interfacial debonding energy, and well-matched ion transport and electrochemical reaction kinetics. Compared with the lithium metal system, sodium metal exhibits a lower yield strength and forms a more brittle SEI, making it more susceptible to localized plastic deformation and interfacial cracking during cycling, thereby accelerating interfacial degradation. Thus, its interfacial stability is more sensitive to electrochemical-stress coupling effects.

#### Thermal–Stress Field Coupling

The combined effects of stress and thermal fields on lithium dendrite formation exhibit a complex pattern. Applying pressure within a suitable range generally suppresses dendrite growth and encourages uniform lithium deposition; however, the influence of temperature is highly material-dependent. For instance, Zaman et al. [112] found that higher temperatures suppress dendritic propagation in LLZO SSEs. This indicates that, for LLZO, combining high temperature with moderate pressure can effectively reduce dendrite formation. On the other hand, high temperatures have been shown to speed up lithium dendrite growth in LPSC [113]. Therefore, in LPSC, the net impact of high temperature and high pressure depends heavily on the balance between these opposing factors.

Based on the micro-mechanism underlying dendritic evolution during the electrochemical deposition of soft metals (e.g., Li, Na), Zhang et al. [114] elucidated the fundamental role of temperature-stress coupling in regulating interface stability and facilitating grain growth. Via multiscale simulations integrated with phase-field modeling and thermodynamic theory, the authors demonstrated that external stack pressure enhances surface energy anisotropy and induces lattice distortion, which favors the deposition of grains with the low-surface-energy [001] orientation. Owing to the elevated diffusion barrier associated with crystal orientation, ion transport becomes constrained, resulting in local strain accumulation that promotes dendrite nucleation and growth. In contrast, increasing the temperature enables dendrite self-suppression and interface self-healing by accelerating atomic diffusion and interface migration, reducing the contribution of strain energy, and shifting the system from a stress-dominated to a diffusion-dominated regime. This thermally induced transition further encourages grain reorientation toward the diffusion-favorable [100] orientation. Moreover, when the temperature and stress are balanced within a critical window, the primary factor influencing grain orientation shifts from energy minimization to kinetic optimization, promoting a dense, uniform deposition morphology.

### Tri-Physical-Field Coupling

Existing dual-physical-field coupling models offer valuable insights into dendrite formation; however, they do not fully capture the complex interactions among the electrochemical, thermal, and stress fields that coexist in operating solid-state batteries. Therefore, a comprehensive investigation into the synergistic influence of strong electrochemical–thermal–mechanical tri-field coupling on dendrite propagation is essential.

**Fig. 14:**
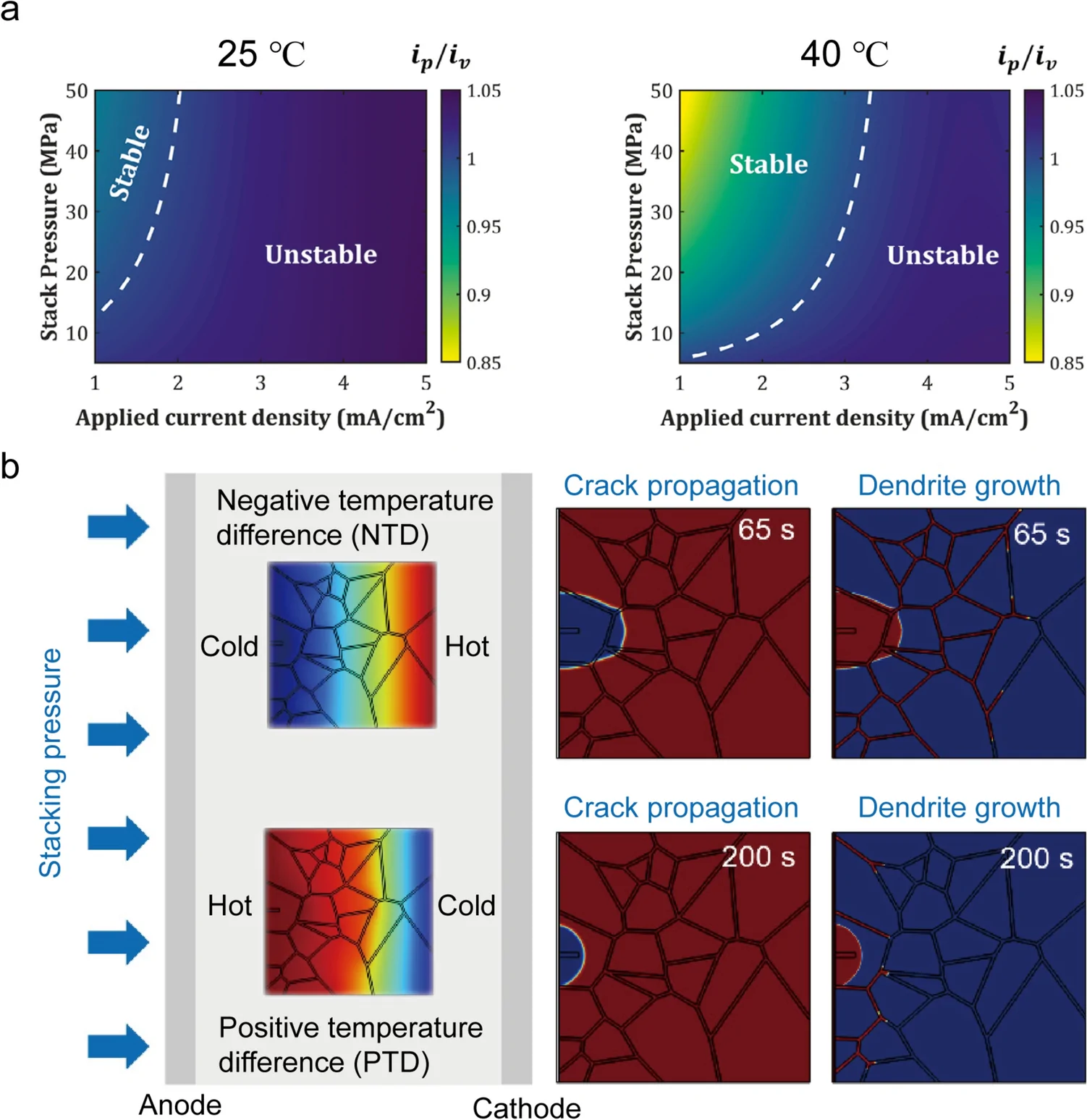
**a** Phase diagram of the influence of stack pressure, temperature, and current density on deposition stability (Reproduced with permission from Ref. [115], Copyright 2024, Wiley–VCH). **b** Effects of temperature gradient and stack pressure on dendrite growth (Reproduced with permission from Ref. [117], Copyright 2025, Tsinghua University Press)

Chatterjee et al. [115] investigated the impact of mechanical, thermal, and electrical fields on dendrite formation. They found that stack pressure induces compressive stress in the concave regions of the anode–electrolyte interface and shifts the mechanical overpotential to more negative values, thereby promoting Li^+^ reduction in these regions. The curvature effect increases the potential gradient in the convex regions, leading to current focusing and dendrite growth. To measure the combined impact of stack pressure, current density, and interface morphology across different temperatures, they introduced the peak-to-valley current ratio [116] (*i*_p_/*i*_v_). As shown in Fig. 14a, at a specific temperature, stress-dominated deposition (*i*_p_/*i*_v_ < 1) inhibits dendrite formation, whereas electrochemistry-dominated deposition (*i*_p_/*i*_v_ > 1) promotes it. An increase in temperature reduces the current focusing effect by improving ion conductivity and exchange current density, which in turn suppresses dendrite growth and broadens the current range for uniform deposition.

In a related study, Pei et al. [117] employed a coupled electrochemical–thermal–mechanical phase-field model to examine the effects of stack pressure, interface defect density, and temperature distribution on lithium dendrite growth. They emphasized the simultaneous development of crack propagation and dendrite formation under uneven thermal conditions. As shown in Fig. 14b, a negative temperature gradient (hotter anode, colder cathode) significantly increases local stress and strain energy density. This creates a concentration gradient that accelerates crack growth, facilitating dendrite penetration through the electrolyte. The application of external pressure under these conditions further worsens crack growth due to the combined effects of pressure and thermal expansion. By contrast, an appropriate level of stack pressure combined with a positive temperature gradient (colder anode, hotter cathode) can suppress dendrite growth, indicating that the temperature-dependent electrochemical driving force plays a more dominant role in dendrite evolution than the mechanical driving force.

Based on the above discussion, the coupled effects of the electrochemical field, thermal field, and stress field essentially manifest as a competitive mechanism between the electrochemical and stress effects for dominance, while the thermal field, serving as a critical auxiliary factor, significantly influences the final behavior of dendrite growth. Specifically, to effectively inhibit dendrite growth, the design of the electrochemical field should favor the stress-dominated deposition mode, ensuring that the *i*_p_/*i*_v_ is less than 1. Accordingly, the mechanical design adapts to the aforementioned stress-dominated deposition mode mainly by precisely regulating stack pressure, avoiding *i*_p_/*i*_v_ > 1 caused by insufficient pressure and preventing electrolyte crack propagation due to excessive pressure. Throughout the entire design process, full consideration should be given to the auxiliary regulatory role of the thermal field. A positive temperature gradient (hotter cathode, colder anode) should be adopted, and the operating temperature should be appropriately increased to enhance ionic conductivity and exchange current density, thereby mitigating the current focusing effect and facilitating dendrite inhibition. It is also worth noting that systematic research on electrochemical–thermal–stress tri-field coupling remains relatively limited, and the above design framework is not universally applicable to all specific battery systems. This represents a key direction for future research efforts.

Furthermore, when extending the above tri-field coupling design framework to sodium battery systems, consideration must be given to their unique inherent features, as summarized in the following three aspects: (1) The relatively low SEI mechanical modulus in sodium batteries changes the state boundary of the stress-dominated deposition mode and impacts the regulatory effect of stress on dendrites. (2) Sodium deposition more often exhibits a surface-growth-dominated morphology, which alters the current focusing behavior at the battery interface and thereby affects the nucleation and growth of dendrites. (3) For sodium battery systems, the optimal pressure window and thermal sensitivity have not yet been fully quantified, leaving an important knowledge gap in this field. These differences imply that modeling sodium battery systems, it is necessary to fully calibrate the mechanical overpotential term, fracture toughness parameters, and temperature-dependent kinetic coefficients to ensure the accuracy and applicability.

In summary, the electrochemical field is the main factor influencing dendrite growth, primarily due to uneven local current densities caused by gradients in electric and ion concentration fields. The thermal field has a dual role: it can promote uniform ion deposition by increasing surface diffusion and reaction kinetics or it can accelerate dendrite development at hot spots. Additionally, the stress field influences this process through mechanical responses such as plastic deformation, creep, and fracture, which alter how dendrites form and grow. The interaction of these physical fields results in more complex phenomena. For instance, the combined effect of thermal and electrochemical fields can lead to a “self-repair” effect at high current densities, in which Joule heating differentially influences energy barriers for electrochemical reactions and ion diffusion. Nonetheless, the most crucial mechanism in SSB failure is the coupling between electrochemical and stress fields. Stress accumulation during repeated electrochemical cycling leads to crack initiation and growth within the solid-state electrolyte. This creates a feedback loop, leading to a cyclical interaction between dendrite growth and crack extension. Ultimately, dendrite formation and growth are governed by the interactions and balance of electrochemical forces, thermally activated kinetics, and mechanical stress under specific operating conditions. Table 2 summarizes the main results from various modeling studies examining these effects, both individually and in combination. To further enhance the understanding of relevant models in solid-state systems, Table 3 provides a comprehensive comparison of their key assumptions, length scales, and limitations.

**Table 2:** Summary of Physical Field Coupling Models

System	Model	Parameters	Conclusion	References
NZSP	Electrochemical–mechanical coupled phase-field model	Grain boundary conductivity and Young’s modulus, polarization potential	The synergistic action of local polarization potential and stress at grain boundaries dominates dendrite penetration	[67]
NZSP	Electrochemical-mechanical coupled model	Current density, creeping stress, crack displacement	Dendrite creep stress is dependent on the local current density	[72]
Carbonate-based	(Modified) Sand’s Time model*	Current density, diffusivity, concentration gradient	Dendrites initiate when the Li^+^ concentration at the interface drops to zero, a process that couples kinetics and diffusion	[77]
				[79]
Ether-based	MD simulation*	Temperature, Li^+^ diffusivity, Nucleation overpotential	At high temperature, Li nucleation overpotential decreases, nucleus size increases, density decreases, and deposition is uniform	[80]
Carbonate-based	Coarse-grained mesoscale model*	Temperature, Li^+^ diffusivity, charge-transfer kinetics	Temperature affects Li deposition by changing Li^+^ diffusion rate, self-diffusion coefficient, and charge transfer exchange current density	[81]
LLZO	Kinetic model	Current density, temperature	CCD increases with temperature	[84]
LPSC	Fracture mechanics model	Stress intensity factor, mechanical energy, crack propagation energy	Dendrite growth is driven by mechanical fracture	[85]
PMMA-based	Linear elastic model	Shear modulus	Dendrites are suppressed when polymer electrolyte shear modulus is more than that of Li medal	[89]
PEO	Elastic–plastic deformation	Plastic deformation, current density, stress relaxation	Dendrite suppression is independent of electrolyte shear modulus, depends on current density ratio at Li peaks/valleys	[90]
				[91]
LLZO	Stress-driven model	Stress gradient, plastic flow, dislocation motion	Stress drives Li flow along grain boundaries	[92]
LPS	Stress model	Overpotential, Li plastic deformation, dislocation climb	Electrodeposition-driven stress induces Li plastic deformation, promoting dendrite growth via dislocation climb and vacancy occupation	[93]
LLZO	MD simulation	Internal stress, structural disorder, elastic modulus	Internal stress accumulation causes SSE fracture, driving dendrite growth	[94]
PEO	Stress-coupled phase-field model	External pressure, chemical potential, interfacial energy	External pressure suppresses dendrite growth above a critical stack pressure	[95]
Carbonate-based	Electrochemical–thermal coupled phase-field model*	Temperature, reaction barrier, SEI formation, Li^+^ transport	Temperature influences dendrite growth via SEI formation and electrochemical deposition	[100]
Carbonate-based	Electrochemical–thermal coupled phase-field model*	Reaction energy barrier, diffusion barrier	Temperature-dependent dendrite growth is controlled by the competition between reaction and diffusion barriers	[101]
Ether-based	Phase-field model*	Activation energy, temperature	The activation energy–temperature relationship governs the promoting, inhibiting, or dual effects on dendrite growth	[102]
LLZO	Electrochemical–mechanical coupled model	Current density, hydrostatic stress, overpotential	Coupled stress effects lead to six dendrite growth mechanisms	[103]
LLZO	Grain boundary resistance theory	Grain boundary conductivity, bulk conductivity	Transgranular, Intergranular	[104]
LLZO	Continuum electro-chemo-mechanical gradient theory	Young’s modulus, Poisson’s ratio, Partial molar volume	Li dendrite growth is intergranular/transgranular with/without stress coupling	[105]
PEO	Stress-coupled Sand’s Time model	Diffusion coefficient	Stress can extend Sand’s time	[106]
LLZO	Electrochemical–mechanical coupled model	Electronic conductivity, CCD	Stress-driven mass transfer diffusion is a component of total current	[107][107]
LLZO	Electrochemical–mechanical coupled model	Fracture toughness, surface energy, overpotential	Kinetic overpotential due to effective stress-induced current accumulation is the root cause of dendrite growth along grain boundary	[54]
LPS				[108]
				[109]
				[110]
LLZO	Thermodynamics-coupled phase-field model	Stack pressure, temperature, lattice distortion energy	Stack pressure enhances lattice distortion and surface energy anisotropy, while higher temperature promotes diffusion shifting the system from stress- to diffusion-dominated	[115]
LLZO	Electrochemical–thermal–mechanical coupled phase-field model	Temperature gradient, electric field gradient, thermal conductivity	Stress and electric field gradient have opposite effects on dendrite, while temperature can suppress current focusing induced by electric field gradient	[116]
LGPS				[118]

Models marked with * are derived from liquid electrolyte systems and are included for fundamental comparison and theoretical basis. All other models are developed or widely applied for solid-state systems

**Table 3:** Comparison of physical field coupling model

Model	Key Assumptions	Scale	Limitations	References
Electrochemical–mechanical coupled phase-field model	Dendrite penetration kinetics are determined by grain boundary conductivity and Young’s modulus and interfacial contact evolution is assumed to have a negligible effect on grain boundary electrochemistry	Meso	Overlooks the dynamic effects of stress relaxation and structural evolution during cycling and fails to simulate dendrite growth transitioning from grain boundaries to the bulk electrolyte	[67]
Electrochemical–mechanical coupled model	Dendrite creep stress is correlated with localized current density	Meso	Lacks quantitative analysis of the quantitative relationship between creep stress magnitude and crack propagation rate	[72]
Kinetic model	Temperature modulates interfacial kinetics by linearly reducing the charge-transfer resistance of the Li-LLZO interface and interface charge-transfer resistance is the dominant factor governing CCD and interfacial electrochemical stability	Meso	Ignores the dynamic evolution of the Li-LLZO interface during long-term cycling	[84]
Fracture mechanics model	Electrochemical reaction kinetics are unaffected by the applied compressive stress and the mechanical strength of SSE is the key parameter governing dendrite-induced failure under applied stress	Meso-Macro	Ignores the possible modulation of applied stress on interfacial ionic transport kinetics and fails to simulate dendrite nucleation behavior, only focuses on post-nucleation propagation	[85]
Linear elastic model	Stack pressure is a core regulatory factor governing the electrochemical-mechanical behavior	Meso-Macro	Ignores the influence of electrolyte microstructural heterogeneity on stress transfer under stack pressure	[89]
Elastic–plastic deformation	Surface tension is not the dominant factor for inhibiting interfacial roughness and dendrite initiation	Meso	Only considers low current density conditions and neglects dendrite behavior at high practical current densities	[90]
				[91]
Stress-driven model	Plating-induced residual compressive stress is the core driving force for lithium dendrite formation and growth	Meso	Does not consider the effect of current density and cycling conditions on stress evolution and dendrite growth	[92]
Stress model	Dendrite-induced stress accumulates at the electrode–electrolyte interface and drives further dendrite propagation	Meso	Ignores the influence of cycling-induced Li stripping on stress relaxation and dendrite morphology evolution	[93]
MD simulation	Lithium dendrite-induced solid electrolyte fracture is governed by the coupling of stress accumulation and electrochemical effects	Micro-Meso	MD simulation framework cannot account for electron transfer/leakage, ignoring electrochemical degradation at grain boundaries	[94]
Stress-coupled phase-field model	External pressure is a dominant regulatory factor for dendrite morphology and growth behavior	Meso	Ignores the synergistic effect of external pressure and temperature/current density on dendrite growth mechanisms	[95]
Electrochemical–mechanical coupled model	Lithium dendrite growth is governed by the intrinsic coupling of electrochemical deposition kinetics and mechanical stress evolution at the electrode–electrolyte interface	Meso	Ignores the influence of microstructural heterogeneities on electrochemical-mechanical coupling	[103]
Grain boundary resistance theory	The polycrystalline conductivity model assumed GBs as isotropic layers, and their conductivity was extrapolated from atomic simulation results	Micro-Meso	The polycrystalline model assumed isotropic GB layers, ignoring the complex distribution and interactions of GBs in actual polycrystalline materials	[104]
Continuum electro-chemo-mechanical gradient theory	The solid electrolyte is treated as a continuum medium, and the damage variable is introduced to describe the microstructural degradation and crack initiation/propagation induced by Li filament growth	Meso	The model assumes a uniform continuum medium for the solid electrolyte, ignoring the inhomogeneity of microstructures in actual ASSB electrolytes	[105]
Stress-coupled Sand’s Time model	Assumes that mechanical stress can alter the Li^+^ diffusion coefficient and electrochemical reaction kinetics at electrode/electrolyte interfaces, and electrochemical lithiation or delithiation can induce volumetric deformation and stress accumulation	Meso	Simplifies the mechanical deformation behavior of solid electrolytes, and may not fully capture the complex fracture, creep and fatigue behaviors of brittle ceramic electrolytes under cyclic stress	[106]
Grain boundary resistance model	The excess electrochemical-mechanical potential driving dendrite formation is counterbalanced by mechanical back stress from dendrite growth within the SSE	Meso	Ignores the effect of SSE microstructural heterogeneities on potential/stress distribution	[107]
Electrochemical-mechanical coupled model	Critical pressure of polycrystalline LLZO correlates with surface energy changes from Li plating at grain boundaries	Meso	Neglects the influence of mechanical stress relaxation on nucleation threshold over time	[108]
Electrochemical–mechanical coupled model	GB fracture is driven by the synergistic effect of stress concentration and current-induced electrochemical degradation	Meso	Focuses on GB-dominated failure, neglecting transgranular dendrite growth and bulk electrolyte fracture mechanisms	[109]
Electrochemical–mechanical coupled model	The severity of dendrite growth correlates directly with the density and distribution of local inhomogeneities in the electrolyte matrix	Meso	Ignores the synergistic effect of external factors on inhomogeneity-induced dendrite growth	[110]
Electrochemical–mechanical coupled model	The coupling effect of stack pressure and mechanical deformation dominates the stability of Li-metal/solid electrolyte interfaces	Meso-Macro	Lack of detailed analysis on the synergistic effect of stack pressure with other factors	[111]
Thermodynamics phase-field model	Stress-induced anisotropy limits kinetics in solid-state Li metal batteries	Micro-Meso	The influence of electrolyte properties on grain selection growth is not discussed	[116]
Electrochemical–thermal–mechanical coupled phase-field model	Assumes Li metal undergoes plastic deformation under stack pressure with negligible hydrostatic stress variation, while SE exhibits heterogeneous hydrostatic stress distribution at rough interfaces	Meso	Simplifies the Li/SE interface as a sinusoidal rough surface and neglects the actual complex interface morphologies	[117]
Electrochemical–thermal–mechanical coupled phase-field model	Crack propagation and lithium dendrite growth in solid-state batteries under abnormal thermal situations are governed by the coupling of thermal expansion, electrochemical driving force, and mechanical stress	Meso	Lacks quantitative analysis of the threshold temperature difference for switching stack pressure’s effect and ignores the synergistic effect of thermal on crack/dendrite cumulative damage	[119]

Micro, Meso, and Macro represent microscale, mesoscale, and macroscale, respectively

## Dendrite-Suppression Strategies in Solid-State Li/Na Metal Battery Systems

**Fig. 15:**
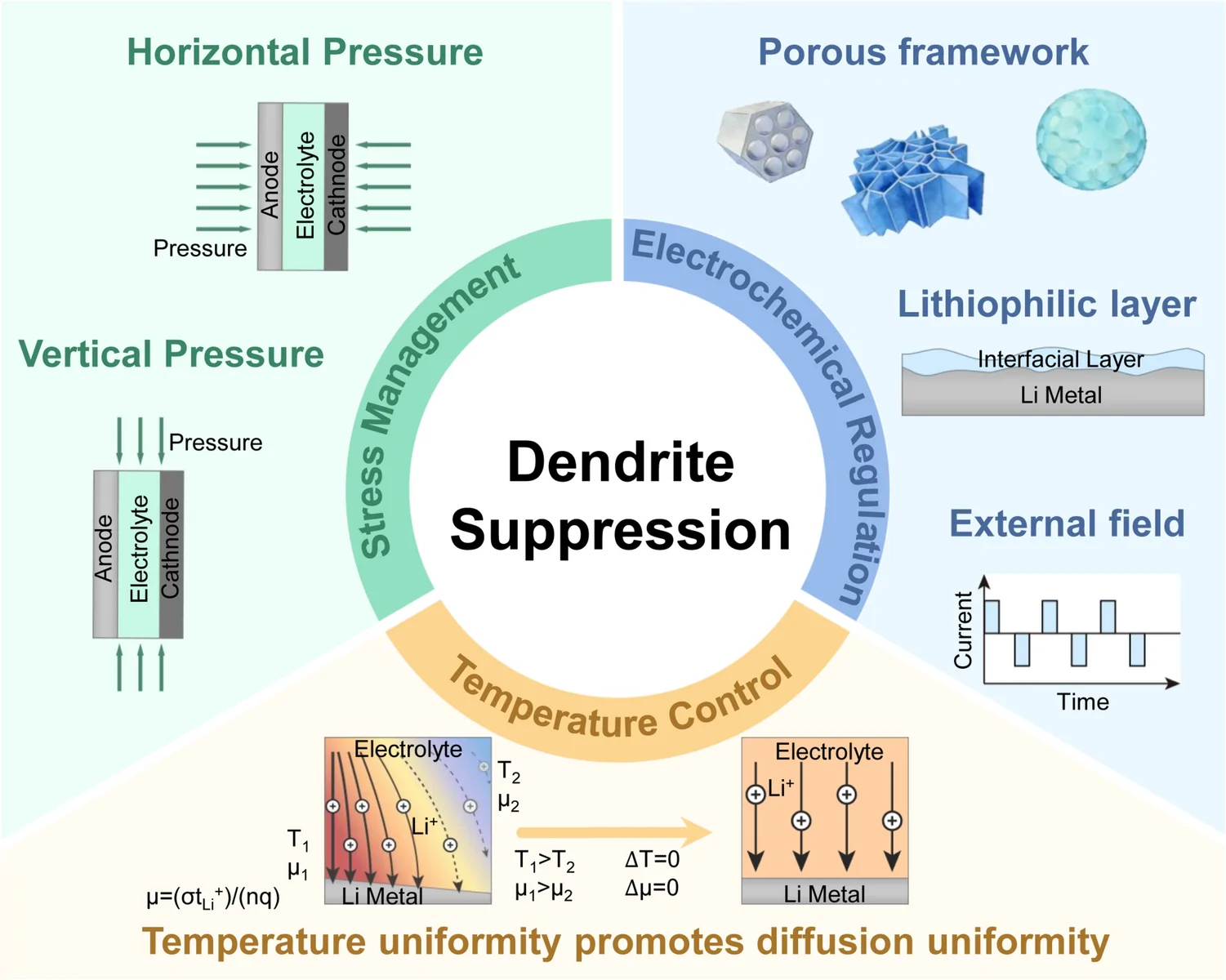
Physics-based strategy for dendrite suppression

Building on the multiphysics framework introduced in Chapter 3, this chapter provides a systematic overview of dendrite-suppression strategies for solid-state Li/Na metal batteries. Unlike traditional classifications based on battery components (such as anode, electrolyte, and interface), this review categorizes existing techniques into three main groups: electrochemical field regulation, thermal field control, and stress field management. The specific strategies are summarized in Fig. 15, and their underlying mechanisms and representative examples are discussed in detail in the following sections.

### Electrochemical Field Regulation

The primary goal of electrochemical field control is to achieve a uniform distribution of ions in space and time, thereby preventing localized deposition that can lead to dendrite growth. This can be achieved through three primary strategies: (1) optimizing interface kinetics, (2) designing ion transport pathways, and (3) applying external fields.

Optimizing the interface kinetics aims to shift the deposition process from diffusion-controlled to reaction-controlled kinetics. This is by enhancing the rate of lithium-ion surface diffusion to surpass the electrochemical reaction rate. For instance, He et al. [118] demonstrated that Sn-alloyed 3D lithium metal anodes exhibit improved cycling stability compared with conventional lithium foil. This enhancement is attributed to the alloying elements, which create abundant lithiophilic nucleation sites and promote surface diffusion. However, this alloying process is energy-intensive because it requires temperatures above the melting points of both lithium and the alloying element. To overcome this, Zhao et al. [119] developed a low-temperature, in situ synthesis method using SbCl_3_ as a precursor and formed a 3D cross-linked structure of LiCl and SbLi_3_, utilizing the principle that bonding high oxidation state cations (Sb^3+^) with Cl^−^ significantly lowers the melting point. The high lithiophilicity and ion conductivity of the structure lead to a significant increase in the critical current density.

Notably, Suo et al. [120] successfully reduced concentration polarization at the electrode–electrolyte interface and removed the impact of the space charge layer by rationally designing an electrolyte (solvent-in-salt electrolyte, SIS [121]) with ultrahigh salt concentration and an exceptionally high lithium-ion transference number. By facilitating the transition from “confined and heterogeneous ion diffusion” to “uniform and high-flux ion transport”, this strategy ultimately led to improved lithium dendrite suppression. This efficient design philosophy derived from liquid electrolytes can be rationally extended to solid-state systems. Chen et al. [122] developed cationic polymer-in-salt electrolytes (PolyIL-IS) to translate the advantages of ultrahigh salt concentration and rapid ion transport into solid-state batteries. By constructing a continuous ion transport network using polymeric ionic liquids and high-concentration lithium salts, these electrolytes enable fast conduction of alkali metal ions through a structural diffusion mechanism, which effectively alleviates concentration polarization and space charge layer effects at solid–solid interfaces. In the preliminary evaluation of sodium-based PolyIL-IS electrolytes, sodium symmetric batteries demonstrated high ionic conductivity and outstanding cycling stability. Through this strategy, the mature ion regulation mechanism originally used in liquid systems can be successfully adapted to solid-state environments, thereby achieving interfacial stabilization and effective lithium dendrite suppression in solid-state metal batteries.

Modifying ion transport pathways, mainly through the design of three-dimensional porous structures and the realization of amorphous grain boundary transformation, is another effective strategy. 3D porous structures offer a large specific surface area, which reduces the effective local current density and promotes uniform ion deposition. Zhang et al. [123] used self-supporting, surface-oxidized 3D hollow porous copper fibers to homogenize lithium-ion flux, effectively confining deposited lithium within the porous structure and enabling stable, high-capacity cycling. Creating an optimal 3D anode requires increasing the specific surface area (*S*_A_) and reducing the structural volume (*S*_V_) [124]. This balance aims to enhance kinetic performance and preserve volumetric energy density, considering the fundamental constraints of electron and ion transport.

**Fig. 16:**
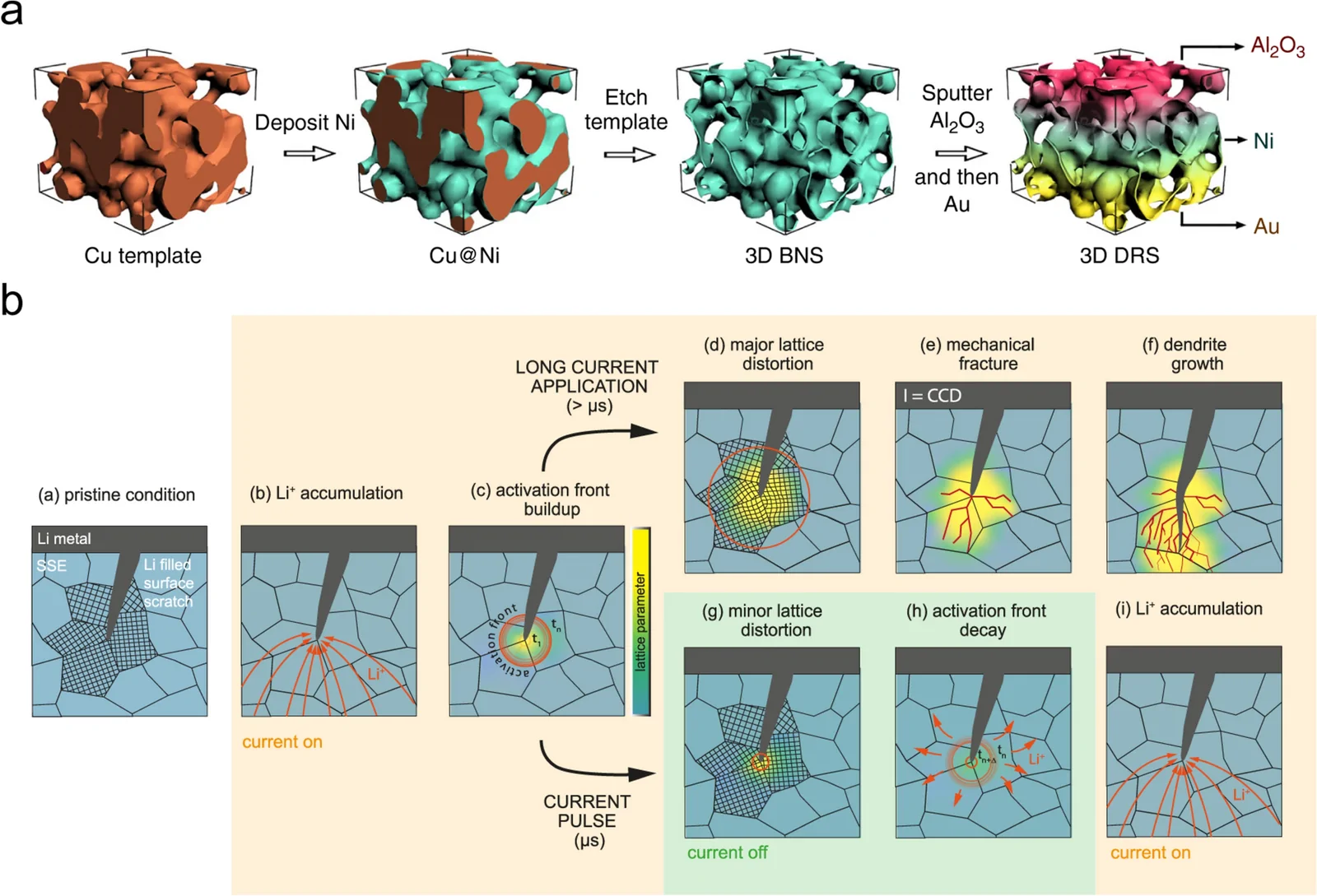
**a** Deposition regulating scaffold composed of Al_2_O_3_-Ni-Au (Reproduced with permission from Ref. [125], Copyright 2019, Springer Nature). **b** Ion accumulation-induced active front (Reproduced with permission from Ref. [133], Copyright 2023, Springer Nature)

By combining this approach with surface modification, as shown in Fig. 16a, Pu et al. [125] developed a deposition regulating scaffold composed of Al_2_O_3_–Ni–Au. This composite structure merges a passivating Al_2_O_3_ layer with a low nucleation barrier Au substrate to encourage uniform lithium deposition through synergistic lithiophilic and conductive effects. 3D structured anodes not only exhibit excellent dendrite-suppression performance in liquid battery systems, but also possess broad application potential in SSB systems. Liang et al. [126] constructed a pressure-tolerant three-dimensional lithium–carbon fiber composite anode, which can effectively homogenize ion flux and realize uniform lithium deposition, thereby inhibiting the nucleation and growth of dendrites, reducing interfacial impedance and mitigating heat generation in the battery, and ultimately enabling stable, safe and short-circuit-free long-term operation of solid-state batteries. Notably, the key to the functionality of this structure lies in its superior mechanical adaptability, which allows it to maintain intimate and stable solid–solid interfacial contact with solid electrolytes even under low assembly pressure. Therefore, it can be noted that the application of 3D structured anodes in solid-state batteries requires focused attention and precise design and regulation of solid–solid interfacial contact and interfacial stability.

Grain boundaries serve as critical pathways for sodium dendrite growth in SSEs, and their intrinsic role is closely associated with ion conduction behavior. To address the critical issues commonly existing in oxide SSEs for solid-state sodium metal batteries, such as high grain boundary impedance, discontinuous ion transport pathways, and the tendency to induce nucleation and growth of sodium dendrites, Yi et al. [127] employed Na_5_SmSi_4_O_12_ as the solid electrolyte and constructed an amorphous grain boundary structure by regulating the non-stoichiometric ratio. This amorphous grain boundary can reconstruct and establish continuous sodium-ion transport pathways, significantly reducing the grain boundary energy barrier and simultaneously improving the bulk and interfacial ionic conductivity. Consequently, the battery system based on this electrolyte achieved stable, dendrite-free cycling for up to 2800h. From the same perspective of reducing crystallinity, Zhao et al. [128] introduced functional molecules to suppress microdomain crystallinity in polymer-based electrolytes. This effectively increased the amorphous ion-conducting regions, enhanced polymer chain mobility and sodium-ion transport efficiency, enabling solid-state sodium batteries based on the modified electrolyte to exhibit excellent cycling stability and rate performance.

External electric and magnetic fields can actively control ion transport and concentration gradients. Applying appropriate pulse currents can improve Li⁺ transport by lowering interfacial resistance and altering the concentration gradient within the SEI [129, 130]. Typically, shorter pulse durations and lower duty cycles help reduce dendrite growth. However, the pulse duration requires careful optimization; it should be longer than the time required to charge the electric double layer (~ 10^−4^ s [131]) but shorter than the time when a Li⁺ depletion layer forms (~ 20 ms [132]). Reisecker et al. [133] observed a sixfold increase in the CCD in polycrystalline LLZTO electrolytes under MHz pulsed currents, with little effect on single-crystal samples. They suggested that pulsing disperses lithium accumulation at defect sites, such as grain boundaries and voids, before a critical front can develop into a dendrite. The higher density of grain boundaries in polycrystalline samples makes them more responsive to this dispersion effect (Fig. 16b). Additionally, Chen et al. [134] demonstrated that AC pulses aligned parallel to the internal electric field, or DC pulses perpendicular to it, can suppress dendrites by promoting Li⁺ migration along the anode surface or by accelerating transport through the electrolyte, respectively.

**Fig. 17:**
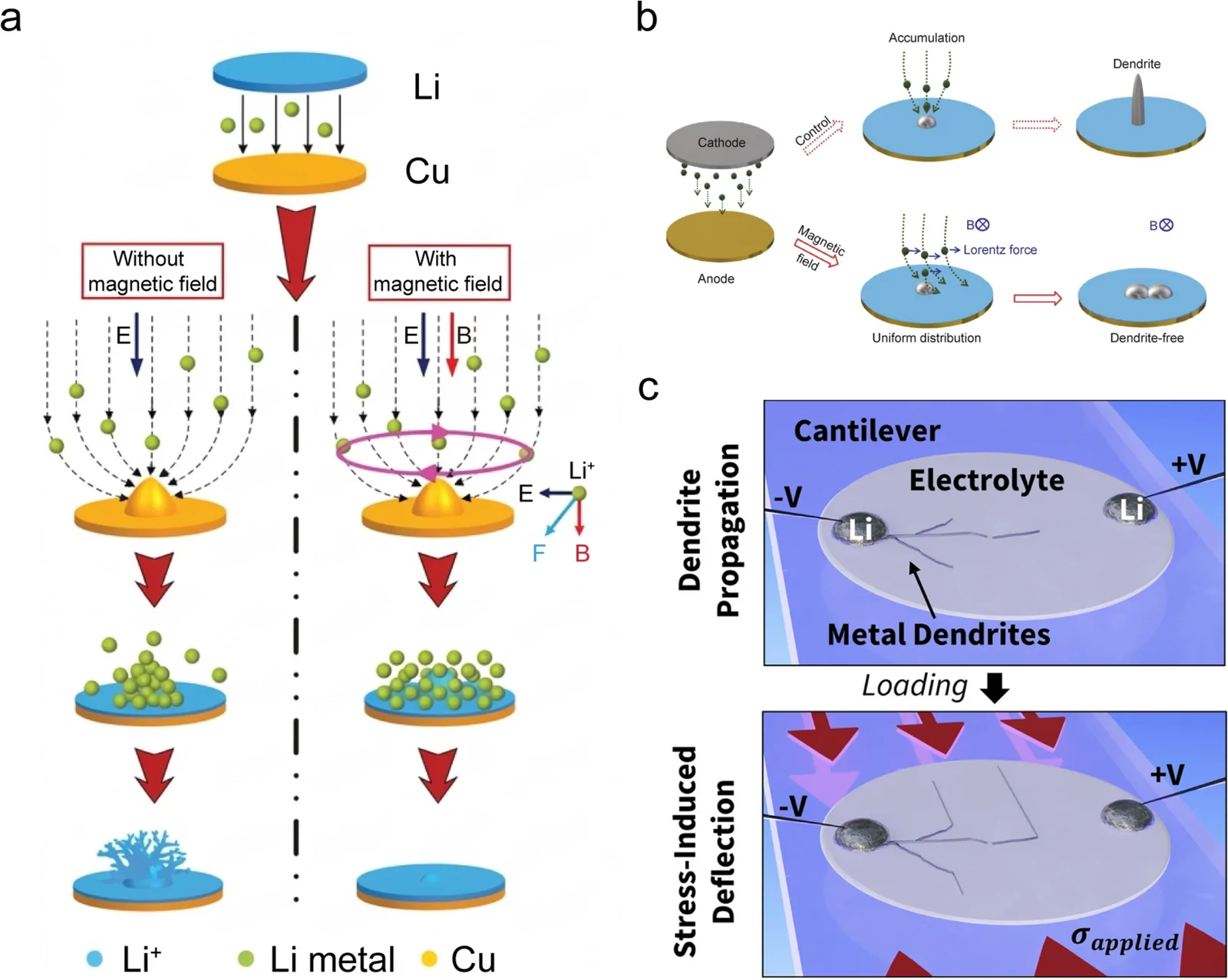
**a** Magnetic field applied parallel. (Reproduced with permission from Ref. [135], Copyright 2019, Wiley–VCH) and **b** perpendicular (Reproduced with permission from Ref. [136], Copyright 2022, Elsevier) to the electric field modulates dendrite. **c** Dendrite deflection under stress (Reproduced with permission from Ref. [84], Copyright 2022, Elsevier)

Magnetic fields have also been employed to control ion mobility via the Lorentz force. Shen et al. [135] applied a magnetic field parallel to the electric field, inducing Li⁺ to follow a spiral path around interfacial protrusions, inhibiting their preferred migration to these sites and supporting uniform deposition (Fig. 17a). Similarly, Chen et al. [136] used a perpendicular magnetic field to generate a Lorentz force that enhances Li⁺ transport along the anode surface and reduces the local concentration gradients that drive dendrite growth (Fig. 17b).

### Thermal Field Control

The influence of the thermal field on dendrite development is dichotomous, while, non-uniform temperature distributions can promote dendritic growth, controlled and uniform heating can be a potent suppression strategy. One mechanism for thermal control is self-heating due to high current densities. Li et al. [25] observed that operating a battery at a high current density not only suppressed dendrite penetration but also improved cycling stability and capacity retention. They attributed this to a self-healing phenomenon: the high overpotential increases nucleation density, and the subsequent Joule heating in high-current–density areas enhances Li^+^ surface diffusion and promotes the fusion of small dendrites, resulting in a smoother electrode surface. Building on this concept, Longchamps et al. [137] developed an internal heating system using ultra-thin nickel foil and a field-effect transistor. This device produces controlled Joule heat, rapidly increasing the ionic conductivity of the electrolyte to encourage uniform lithium deposition, especially at low ambient temperatures.

Proactive thermal management also fundamentally alters lithium nucleation behavior. Yan et al. [82] demonstrated that increasing the operating temperature to 60 °C resulted in a lower nucleation density but significantly larger lithium nuclei. These larger, sparsely distributed nuclei then grow and merge into a dense, flat lithium layer, effectively preventing mossy or dendrite structures. In addition to in situ operational control, thermal treatment can also serve as a post-mortem repair method. Chen et al. [138] applied thermal annealing to LLZTO SSEs that had previously failed due to dendrite growth. They found that this process restored the electrolytes by increasing their relative density and ionic conductivity. Further analysis revealed that heating causes the reaction byproducts of lithium dendrites with air (LiOH, Li_2_CO_3_) to act as sintering aids. These compounds fill microstructural defects, such as pores and grain boundaries, making the electrolyte denser and more resistant to dendrite growth.

Notably, Yu et al. [139] proposed a strategy to effectively suppress dendrite growth by applying a through-thickness thermal gradient to LLZTO solid electrolytes. Related experiments demonstrate that a moderate temperature difference of only 20 °C could increase the CCD by nearly three times and extend the cycle lifespan by more than four times. In essence, the core of this strategy lies in inducing internal compressive stress via the thermal gradient, representing a mechanical regulation method based on the coupling of thermal and stress fields. This finding also implies that it is hard to strictly decouple individual physical fields in practical battery systems, where field coupling effects are ubiquitous and play a critical role in determining interfacial stability.

Thermal field control can also be applied to the preparation of battery materials. Zou et al. [48] proposed a nanotwinned-alloy strategy based on Joule heating treatment. In this strategy, the instantaneous, non-equilibrium extreme thermal field generated by Joule heating enables the rapid and uniform segregation of silicon phases from supersaturated solid solutions and induces the formation of nanotwinned structures with high-density twin boundaries. The unique microstructure precisely regulated by the thermal field significantly improves Na^+^ interfacial transport kinetics and deposition uniformity, successfully converting the sodium deposition mechanism from diffusion-controlled to reaction-controlled and achieving a highly stable, dendrite-free sodium metal anode.

### Stress Field Management

Unlike liquid electrolytes, the rigidity of SSEs hinders the effective relaxation of deposition-induced stress, leading to stress concentration and mechanical failure, key drivers of dendrite propagation. Consequently, managing the stress field represents a critical and unique challenge in SSB design. Gao et al. [140] addressed the critical issues of insufficient mechanical strength and susceptibility to sodium dendrite penetration in Na_3_Zr_2_Si_2_PO_12_ (NZSP) solid electrolytes by introducing TiO_2_ as a second phase into the NZSP matrix to enhance mechanical performance. Through the dispersion strengthening effect of TiO_2_, this strategy significantly improves the densification and grain boundary bonding strength of the electrolyte, reduces internal defects, and increases the shear modulus from 3.5 to 5.2 GPa, which satisfies the Monroe–Newman mechanical suppression criterion, thus providing physical resistance against sodium dendrite penetration from a mechanical perspective.

In most scenarios, a primary strategy for stress field control is the application of external stack pressure. Feng et al. [141] found that applying sufficient stack pressure promotes voids to migrate away from the anode–electrolyte interface, thus enhancing interfacial contact. However, for this to be effective, the applied pressure must exceed the critical stack pressure [95] to trigger lithium creep, which offsets dissolution and prevents void formation. This pressure must be balanced against the fracture strength of the material to avoid cracking and the development of electrically isolated “dead lithium”.

Beyond suppression, stack pressure can also be employed to modify the dendrite propagation pathway. As shown in Fig. 17c, Fincher et al. [84] demonstrated that, in the absence of an external force, lithium dendrites typically penetrate the electrolyte linearly along a direction perpendicular to the electrode surface, forming characteristic straight-through penetration cracks. However, when sufficient stack pressure is applied, the propagation direction shifts from perpendicular to oblique, aligning with the path of maximum shear stress. This adjustment results in a more tortuous, zigzag path within the electrolyte, significantly prolonging the time needed to cause a short circuit.

Dynamic stress control offers an alternative to static pressure. Ye and Li [142] proposed a multilayer electrolyte with a “sandwich” structure designed to generate in situ compressive stress via an “expansion bolt” effect. In their design, an electrochemically less stable inner layer (LGPS) is placed between two more stable outer layers (LPSC). As a dendrite propagates, it induces localized decomposition of the inner LGPS layer. The resulting reaction byproducts undergo volumetric expansion, creating a localized compressive field that arrests further crack and dendrite growth. Finally, stress can be managed by engineering the mechanical properties of the anode substrate. Wang et al. [91] observed that rigid copper current collectors promote the formation of sharp, acicular dendrites, whereas a compliant, deformable substrate facilitates the growth of uniform, rounded lithium deposits. Based on this principle, they developed a 3D flexible porous scaffold (3D Cu@PDMS) with a polydimethylsiloxane skeleton. The low elastic modulus of PDMS enables the scaffold to deform in response to compressive stress induced by deposition. This mechanism dissipates stress across the structure, preventing the localized stress concentrations that typically initiate dendrite formation and enabling highly stable cycling at high current densities. Similarly, Yan et al. [143] designed and fabricated a porous reduced graphene oxide (PRGO) film with both flexibility and sodiophilicity as the substrate for sodium metal deposition. The flexible substrate effectively relieves interfacial contact strain through adaptive deformation, which significantly mitigates stress concentration and structural distortion of the electrode. By maintaining interfacial stress at a low level, it promotes dense and uniform sodium deposition, resulting in a dendrite-free sodium metal anode.

## Summary and Perspective

This review provides a comparative analysis of dendrite nucleation, growth, and morphology in liquid and solid-state Li/Na metal batteries, while systematically summarizing the distinct yet interconnected roles of electrochemical, thermal, and stress fields. A key insight is that dendrite formation in solid-state systems depends on a more complex set of factors, including interfacial contact, grain boundary networks, and microstructural porosity, all of which are critically important for nucleation and growth. In particular, the significance of grain boundaries and preexisting cracks highlights the strong coupling between mechanical and electrochemical fields in driving mechanical failure. Although current research has advanced the understanding of dendrite formation in solid-state batteries, significant challenges still remain. Key limitations include the lack of quantitative modeling of multiphysics interactions, limited capability for real-time observation of dynamic processes, and limited mechanistic understanding of complex interfacial environments. To address these challenges, future research should focus on the following directions:

1. Advancing quantitative in situ characterization of multiphysics phenomena. Although current techniques enable effective visualization of dendrite nucleation and growth, a significant challenge lies in the in situ quantification of key physical parameters under coupled multiphysics conditions. Future efforts should move beyond qualitative dynamic observations toward quantitative, cross-scale parameter measurements to systematically capture the correlations between physical field evolution and dendrite behavior across multiple length scales.
2. Development of cross-scale, three-dimensional models. Most existing models are simplified to two dimensions, which fails to capture the inherent 3D nature of a battery system. The anisotropy of material properties, particularly in SSEs, governs the distinct three-dimensional distribution and evolution of physical fields. Therefore, the development of 3D coupled models that incorporate multiscale, anisotropic features spanning from the atomic to the cell and pack level is essential for accurate predictions.
3. Elucidating tri-field coupling mechanisms. The interaction of physical fields in solid-state batteries is more complex than in liquid systems. To date, research has mainly focused on single physical fields or dual-field electrochemical-stress coupling. Future studies need to investigate the fully coupled effects of electrochemical, thermal, and stress to understand the governing principles of dendrite growth under combined influence conditions.
4. Multiphysics-guided materials design. The application of a multiphysics framework should extend beyond fundamental understanding to actively guide the rational design of advanced materials. This underscores a shift toward engineering material properties based on their response to coupled physical fields. A critical next step is to combine high-throughput computation, data-driven analysis, targeted material synthesis, and multiphysics modeling to create a feedback loop that efficiently discovers and optimizes dendrite-resistant materials.
5. Establishing standardized benchmarks and constructing comprehensive data systems. The establishment of standardized benchmark experimental protocols, integrated with the development of comprehensive supporting databases, is essential for advancing multiphysics research toward reproducibility and quantitative validation. We advocate for the adoption of standardized battery geometries and unified multiphysics testing protocols. For example, CCD measurements should be conducted under well-defined heating rates and externally applied stack pressures, with systematic recording of key descriptors such as temperature gradients, stress evolution, and interfacial contact variations. Implementing unified testing specifications and structured data formats will significantly enhance cross-laboratory comparability, scientific reproducibility, and model verification efficiency.

Furthermore, research on solid-state sodium metal batteries still remains significantly less developed than that on lithium-based systems. Owing to the intrinsic differences between sodium and lithium in diffusion kinetics, interfacial reactivity, and the composition and mechanical properties of the SEI, direct transplantation of lithium-derived models and parameters is often inadequate. Dedicated and systematic investigations tailored to sodium systems are urgently required, including the development of specialized databases and coordinated experimental-modeling studies.
